# Factors controlling surface oxygen exchange in oxides

**DOI:** 10.1038/s41467-019-08674-4

**Published:** 2019-03-22

**Authors:** Yipeng Cao, Milind J. Gadre, Anh T. Ngo, Stuart B. Adler, Dane D. Morgan

**Affiliations:** 1 Department of Material Science and Engineering, University of Wisconsin–Madison, Madison, WI 53706 USA; 2 Argonne National Laboratory, Lemont, IL 60439 USA; 3 Department of Chemical Engineering, University of Washington, Seattle, WA 98195 USA

## Abstract

Reducing the working temperature of solid oxide fuel cells is critical to their increased commercialization but is inhibited by the slow oxygen exchange kinetics at the cathode, which limits the overall rate of the oxygen reduction reaction. We use ab initio methods to develop a quantitative elementary reaction model of oxygen exchange in a representative cathode material, La_0.5_Sr_0.5_CoO_3−δ_, and predict that under operating conditions the rate-limiting step for oxygen incorporation from O_2_ gas on the stable, (001)-SrO surface is lateral (surface) diffusion of O-adatoms and oxygen surface vacancies. We predict that a high vacancy concentration on the metastable CoO_2_ termination enables a vacancy-assisted O_2_ dissociation that is 10^2^–10^3^ times faster than the rate limiting step on the Sr-rich (La,Sr)O termination. This result implies that dramatically enhanced oxygen exchange performance could potentially be obtained by suppressing the (La,Sr)O termination and stabilizing highly active CoO_2_ termination.

## Abstract (web summary)

The performance of solid oxide fuel cells relies on oxygen exchange kinetics, which can limit oxygen reduction at the cathode. Here the authors use ab initio methods to model oxygen exchange in a representative cathode material to better understand the active molecular mechanism toward optimized design.

## Introduction

**Fig. 1:**
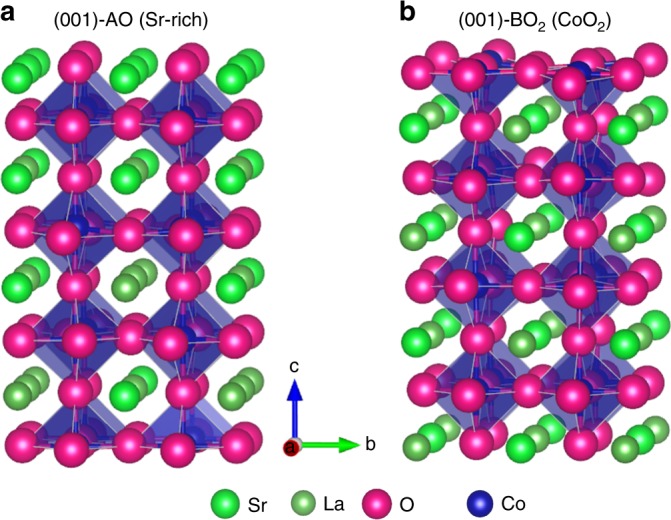
La_0.5_Sr_0.5_CoO_3_ (LSC-50) terminations. **a**. Schematic of the 8-layered surface slab terminated with Sr-rich (001)-AO (SrO) for LSC-50. This surface is referred to as ‘Sr-rich AO’ or simply ‘SrO’ termination in the text. Elements are represented as spheres with ionic radii: La^3+^ (dark green), Sr^2+^ (light green), Co^3+^(purple, center of the octahedra, not seen in this projection), and O^2−^ (magenta). **b**. Schematic of the 8-layered surface slab terminated with (001)-BO_2_ (CoO_2_) of LSC-50. Details of the simulation setup are in the “Methods” section

In solid oxide fuel cell (SOFC) cathodes, which are the primary motivation for this oxygen exchange study, the forward process of oxygen exchange is oxygen reduction reaction (ORR) that takes gas phase O_2_ and transforms it to solid phase O^2−^ in the cathode. The exchange is comprised of O_2_ adsorption, dissociation, and incorporation at the surface, followed by O^2−^ diffusion in the bulk^1,2^. SOFC cathode materials that perform these operations efficiently are almost all mixed electronic and ionic conducting complex oxides, and typically have the perovskite structure (Fig. 1) with stoichiometry ABO_3-δ_ (where A and B are generally metal and transition metal elements, respectively). In spite of many experimental^3–9^ and modeling^1,10–15^ efforts, a quantitative molecular understanding of oxygen exchange at the surface of mixed conducting oxides remains elusive. This limited understanding means one cannot presently predict which materials or surfaces will be most active for exchange and inhibits rational design of optimal materials.

Previous ab initio studies^14–20^ have investigated the nature of stable surfaces and adsorbates on SOFC cathodes, but typically without developing quantitative models for oxygen exchange and usually with a limited focus on the (001) BO_2_ terminated surface (Fig. 1b). However, recent work strongly suggests that Sr-doped perovskites such as La_1−x_Sr_x_MnO_3−δ_ (LSM)^21^, La_1−x_Sr_x_CoO_3−δ_ (LSC)^22–26^ and La_1−x_Sr_x_Co_1−y_Fe_y_O_3−δ_ (LSCF)^27^ all have Sr enrichment in the surface, which indicate Sr segregation and/or precipitation of the second phase. Furthermore, experiments^23,25,27,28^ and ab initio studies^29^ show some specific evidence for stabilized Sr-rich (La,Sr)O (or AO) terminations, demonstrating the need to understand the exchange process on the AO surface. Recent work on (La,Sr)CoO_3_ cathodes in particular has also shown significant oxygen exchange rate degradation within hours of operation^24,30^, strong Sr segregation^22,23,26,31,32^, reversal of degradation after chemical etching^33^ in Sr-doped cathode materials, and a major role for a small number of highly active Co sites in the oxygen exchange rate of the AO surface^34^. The coupling of these chemical and performance changes cannot be understood without a detailed model for the oxygen exchange. A recent semi-quantitative work by Mastrikov et al.^15^ predicts an approximate 5 orders of magnitude difference of exchange rate between MnO_2_ termination and (La,Sr)O termination in LSM, although without a comprehensive kinetic model. There is therefore a strong need to develop better understanding of the atomic scale mechanisms controlling the oxygen exchange.

Here we combine ab initio (Density Functional Theory) reaction energetics, defect chemistry and microkinetic modeling to calculate and compare absolute rates for 53 different mechanisms of oxygen exchange (see “Results” section) on both AO (Fig. 1a) and BO_2_ (Fig. 1b) surfaces of La_0.5_Sr_0.5_CoO_3−δ_ (LSC-50), a representative transition metal perovskite cathode for SOFCs. We note that this is a simplified model that leaves out many possible complexities, including other active surface orientations, possible roles for steps/corners and other undercoordinated surface sites, and the impact of possible additional phases at the surface (e.g., SrO_x_ and Ruddlesden–Popper phases^35^). In particular, we cannot be sure that the assumption for SrO termination will be satisfied by the materials used in the experiments^5,7–9^ to which we compare (see “Results” section). However, there are many experiments^23,25,27,28^ and ab initio studies^29^ that strongly support our assumption, so we think it is a reasonable one. While the present model provides valuable insights and good agreement with available experiments, we do not mean to suggest that this model provides the definitive mechanistic understanding for all possible conditions for LSC oxygen exchange.

## Results

### Thermodynamic and kinetic model

To quantify the activity of a surface towards oxygen exchange we will follow common practice and use the surface exchange coefficient, *K*_tr_, which is defined as the proportionality between the rate of oxygen incorporation, *r*, and deviation of surface oxygen concentration $\left( {\left[ {{\mathrm{O}}_{{\mathrm{O}},{\mathrm{S}}}^{{X}}} \right]} \right)$, from equilibrium $\left( {\left[ {{\mathrm{O}}_{{\mathrm{O}},{\mathrm{S}}}^{{{x}},{\mathrm{eq}}}} \right]} \right)$, through the relationship, $r = - K_{\mathrm{tr}}\left( {\left[ {{\mathrm{O}}_{{\mathrm{O}},{\mathrm{S}}}^{{X}}} \right] - \left[ {{\mathrm{O}}_{{\mathrm{O}},{\mathrm{S}}}^{{{x}},{\mathrm{eq}}}} \right]} \right)$. We will also make use of a more fundamental parameter, the equilibrium surface exchange rate, *R*_0_, which gives the amount of oxygen per unit area and time that is exchanged between the material and gas at equilibrium and is easily related to *K*_tr_ (see the Rate expressions section in the “Methods” section and ref. ^11^).

In this work, kinetic rate expressions for individual ORR mechanisms are formulated assuming a particular step of the mechanism being rate-limiting, implying that the overall driving force (which equals the total free energy change for the reaction of O_2_ getting incorporated into bulk of the cathode ${\mathrm{O}}_2 +{{2\mathrm{V}}}^{..}_{{\mathrm{O}}_{\mathrm{b}}}+ {\mathrm{4e}}^ - \leftrightarrow {\mathrm{2O}}_{{\mathrm{O}}_{\mathrm{b}}}^{{X}}$,) equals the free-energy change in the specific rate-limiting elementary step^11^. To describe the thermodynamics of various elementary steps of ORR we use a neutral building-unit representation of reactants and products, which facilitates the formulation of chemical potentials and description of mechanisms. These are defined in Supplementary Table 1 (SrO surface) and Supplementary Table 4 (CoO_2_ surface). For example, neutral building units ‘s’, ‘sO_2_’, ‘CoO’ denote surface oxygen vacancy, O_2_ inserted into surface vacancy, and O-adatom on surface Co, respectively. We use Kröger–Vink notations for defects. For more details of the thermo-kinetic framework used in this work please refer to Adler et al.^11^.

As a specific example, we describe one of the mechanisms on CoO_2_ termination and then formulate the rate expressions (refer to “Methods” section for the rate expression). The same framework has been used for all the other mechanisms. The following describes Mechanism B3 of Supplementary Table 6, which involves adsorption at an oxygen vacancy.

**Step 1**, O_2_ adsorption on a vacancy and dissociation by insertion into the vacancy, (2s + O_2_ → sO_2_ + s(far)) or,

1a $$2{{\mathrm{V}}}_{{\mathrm{O}}_{\mathrm{S}}}^{q_{\mathrm{S}}^.} + {\mathrm{O}}_{{\mathrm{2,gas}}} + q_{{\mathrm{ads}}}{\mathrm{e}}^ - + q_{{\mathrm{diss}}}{\mathrm{e}}^ - \leftrightarrow \left( {{\mathrm{O}}_{{\mathrm{O}}_{\mathrm{S}}}^{q_{{\mathrm{O}}_2}^.} - {\mathrm{O}}_{{\mathrm{O}}_{\mathrm{S}}}^{q_{\mathrm{O}}^.}} \right)$$

Here, *q*_ads_ denotes charge transferred to oxygen from the LSC surface during adsorption, *q*_diss_ denotes charge transferred to oxygen from the LSC surface during insertion into nearby vacancy, regular brackets on product side indicate O–O configuration inserted into a vacancy on the CoO_2_ surface (denoted sO_2_ in neutral building unit notation),

**Step 2**, Diffusion of *O towards surface vacancy (sO_2_ + s(far) → CoO + s(near)) or,

1b $${\mathrm{O}}_{{\mathrm{O}}_{\mathrm{S}}}^{q_{{\mathrm{O}}_2}^.} + {{\mathrm{V}}}_{{\mathrm{O}}_{\mathrm{S}}}^{q_{\mathrm{S}}^.} + q_{{\mathrm{diffusion}}}{\mathrm{e}}^ - \to {\mathrm{O}}_{{\mathrm{Co}}}^{q_{{\mathrm{O}}_2}^.} + {{\mathrm{V}}}_{{\mathrm{O}}_{\mathrm{S}}}^{q_{\mathrm{S}}^.}$$

**Step 3**, Incorporation into the surface, (CoO + s(near) → null) or,

1c $${\mathrm{O}}_{{\mathrm{Co}}}^{q_{{\mathrm{O}}_2}^.} + {{\mathrm{V}}}_{{\mathrm{O}}_{\mathrm{S}}}^{q_{\mathrm{S}}^.} + q_{{\text{surface-incorp}}}{\mathrm{e}}^ - \to {\mathrm{O}}_{{\mathrm{O}}_{\mathrm{S}}}^{q_{\mathrm{O}}^.}$$

**Step 4**, Incorporation into the bulk LSC-50: (v → s) × 2 or,

1d $${\mathrm{O}}_{{\mathrm{O}}_{\mathrm{S}}}^{q_{\mathrm{O}}^.} + {{\mathrm{V}}}_{{\mathrm{O}}_{\mathrm{b}}}^{..} + q_{{\text{bulk-incorp}}}{\mathrm{e}}^ - \to {\mathrm{O}}_{{\mathrm{O}}_{\mathrm{b}}}^{{X}} + {{\mathrm{V}}}_{{\mathrm{O}}_{\mathrm{S}}}^{q_{\mathrm{S}}^.}\left( {{\mathrm{x}}2} \right)$$

Where *q*_diffusion_, *q*_surface-incorp_, *q*_bulk-incorp_ are charge transfer involved in diffusion, surface incorporation and bulk incorporation of oxygen respectively. The “Methods” section details the calculations of exchange rates based on DFT energetics.

**Fig. 2:**
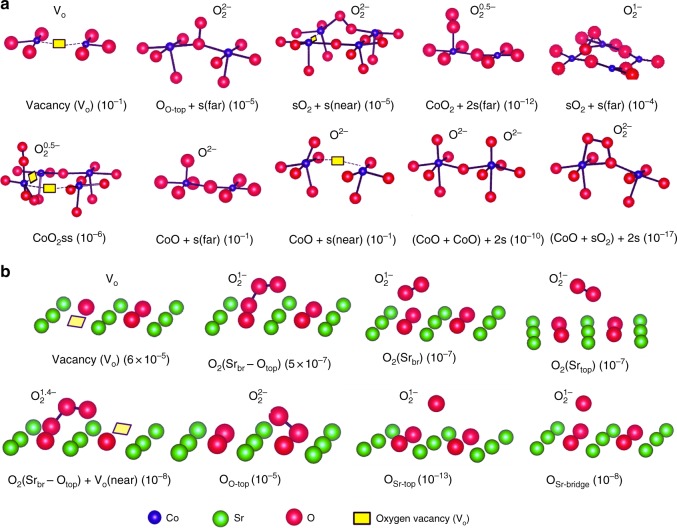
Different ORR intermediates on the (001)-SrO-termination and the CoO_2_ termination. **a** These include the products of oxygen adsorption and oxygen dissociation and oxygen incorporation reactions on the SrO terminated LSC surface. Concentrations (per-Co site) of surface species at 650 °C and 0.2 atm *p*O_*2*_ are given in parentheses. ${\mathrm{O}}_2^{{n} - }$ represent adsorbed oxygen molecular with number *n* negative electron charge on the surface. O_top_, O_**Sr-top**_ and O_**Sr-bridge**_ represent an adatom oxygen at oxygen top, Sr top and Sr bridge position respectively. O_2_(Sr_top_), O_2_(Sr_br_) and O_2_(Sr_br_ − O_top_) represent a adsorbed O_2_ at Sr top, Sr bridge and between Sr bridge and O top, respectively. **b** These include the products of oxygen adsorption and oxygen dissociation and oxygen incorporation reactions on the CoO_2_ terminated LSC surface. Concentrations (per-Co site) of surface species at 650 °C and 0.2 atm *p*O_*2*_ are given in parentheses. “s” represents a surface oxygen vacancy. CoO represent an oxygen adatom on the top of surface Co atom. “sO_2”_ represent an oxygen molecular incorporated into a surface oxygen vacancy

**Fig. 3:**
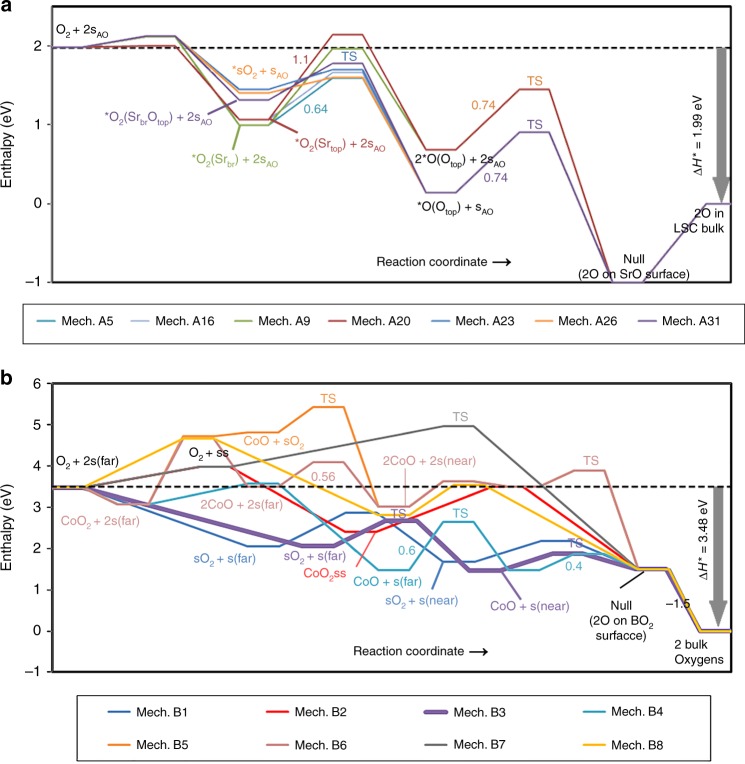
(001)-SrO terminated surface energy landscape and CoO_2_(001) terminated surface energy landscape. **a** The energy landscape for oxygen adsorption, dissociation, and incorporation into the SrO (001) terminated surface and the bulk of the La_0.5_Sr_0.5_CoO_3_ (LSC-50) perovskite. Energy on *y*-axis is the DFT calculated enthalpy with correction. The initial configuration is O_2_ molecular and two isolated surface vacancies (2s_AO_) on the SrO-surface, final configuration is ‘null’ surface, followed by 2-O in the bulk of LSC-50, all three states being common for all mechanisms. The overall change in the enthalpy is denoted by ΔH* = 1.99 eV. Different mechanisms are described in the “Methods” section and the Supplementary Note 1. Only seven kinetically mechanisms are shown. All reaction intermediates are denoted in respective colors and underlined. Transition states are shown as ‘TS’, with the forward reaction barriers (in eV) are written next to the step proceeding through a transition state, or in parenthesis for clarity. **b**. The energy landscape for O_2_ adsorption, dissociation and incorporation into the CoO_2_(001) terminated surface and finally into the bulk of the LaSrCoO_3_ (LSC-50) perovskite. Energy on *y*-axis is the DFT calculated enthalpy with correction. The initial configuration is O_2_ molecular and two isolated surface vacancies (2s, or s + s) on the LSC surface, final configuration is ‘null’ surface (2-O on surface), followed by ‘bulk’ (2-O in bulk), all three states being common for all mechanisms. Overall change in the enthalpy is denoted by ΔH* = 3.48 eV as shown. Different mechanisms are described in “Methods” section and the Supplementary Note 1. Direct dissociative chemisorption mechanisms (Mechanisms B9 through B12) are not included in this figure as they have large chemisorption energy barriers that make the mechanisms orders of magnitude slower than all other mechanisms. All reaction intermediates are denoted in respective colors and underlined. The symbol ‘far’ denotes isolated species whereas ‘near’ denotes species occupying nearest-neighbor surface sites. Transition states are shown as TS. Mechanism B3 (thick purple line) emerges as the fastest ORR mechanism on BO_2_ termination with O_2_ adsorption and dissociation as rate-limiting step

DFT-predicted molecular configurations of various ORR intermediates (products of oxygen adsorption dissociation, incorporation) are depicted in Fig. 2a (SrO termination) and in Fig. 2b (CoO_2_ termination). Based on the DFT-predicted Gibb’s free energy relative to O_2_ gas, the concentrations of these species is also indicated at relevant SOFC conditions (oxygen partial pressure *p*O_*2*_ *=* 0.2 atm, temperature *T* = 650 °C) in these figures as well as Supplementary Table 8. Many distinct (surface site-specific) reaction mechanisms may proceed through these surface intermediates, each potentially contributing to the overall ORR reaction of O_2_(gas) incorporation into the bulk oxide. For convenience, these mechanisms are classified here by their initial adsorption step and the consequent dissociation step(s). In this way, 41 distinct mechanisms emerge on the SrO termination (see Supplementary Table 2) and 12 mechanisms on CoO_2_ termination (see Supplementary Table 5). The ORR is unlikely to proceed through the mechanisms for which the surface intermediate concentrations are predicted to be too low (such as CoO_2_ + 2s(far) intermediate, see Fig. 2b), whereas more stable surface intermediates (such as s, CoO + s(far) on CoO_2_ surface, O_O-top_ on SrO surface) would be expected to appear in the most prominent (fastest) mechanisms, since kinetic rates generally scale with concentration of (reactant) surface intermediates. Figure 3 show the DFT-predicted energy landscape of key mechanisms on SrO and CoO_2_ terminations, respectively. The *y*-axis of Fig. 3 is DFT corrected enthalpies (relative to O_2_ molecular and isolated vacancies on the oxide surface), where the corrections are described in the DFT Corrections section of the “Methods” section. The initial step in the energy landscape of all mechanisms is O_2_(gas) + 2s, whereas the final step is two oxygen ions incorporated in the bulk.

### Surface oxygen exchange activity

**Fig. 4:**
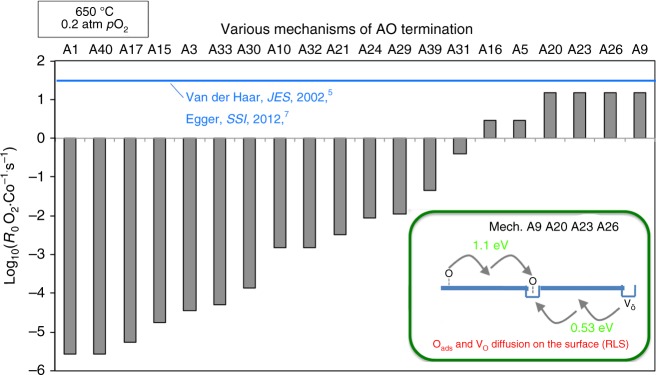
SrO-terminated surface exchange rate for different mechanisms. Comparison of various ORR mechanisms on the SrO-terminated LSC-50 surface, in terms of surface oxygen exchange rates (log_10_(*R*_0_, # O_2_·Co^−1^·s^−1^)) predicted at *T* = 650 °C and *p*O_*2*_ = 0.2 atm. The unit O_2_·Co^−1^·s^−1^ is number of adsorbed oxygen molecular at one surface Co area per second. Blue vertical line is the average *R*_0_ derived from the reported experimental exchange rate coefficients (*K*_tr_, m·s^−1^) by van der Haar et al.^5^ and Egger et al.^7^ for LSC-50 and at the same conditions of temperature and pressure. Molecular schematics are provided, with rate-limiting step indicated as ‘RLS’. Mechanisms A9, A20, and A23, A26 emerge as the fastest, and agrees well with the experimental values

**Fig. 5:**
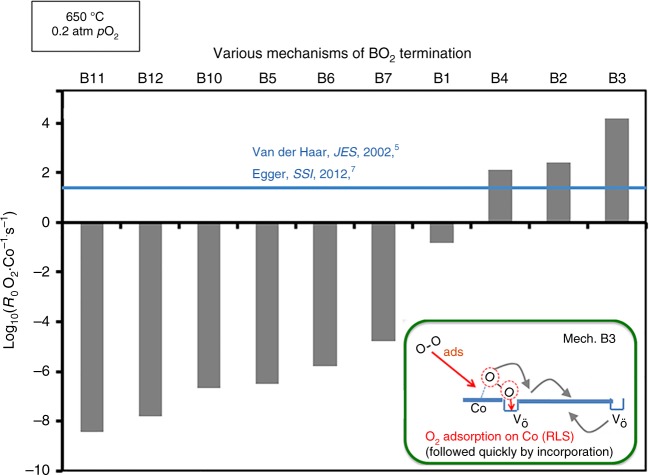
CoO_2_-terminated surface exchange rate for different mechanisms. Comparison of various ORR mechanisms on the CoO_2_-terminated LSC-50 surface, in terms of surface oxygen exchange rates (*R*_0_, # O_2_·Co^−1^·s^−1^, plotted in log-scale) calculated at *T* = 650 °C and *p*O_*2*_ = 0.2 atm. Blue vertical line is the average of *R*_0_ derived from the reported experimental exchange rate coefficients (*K*_tr_, m·s^−1^) by van der Haar et al.^5^ and Egger et al.^7^ for LSC-50 and at the same conditions of temperature and pressure. Molecular schematics are provided, with rate-limiting step indicated as ‘RLS’. Mechanism B3 (O_2_ chemisorption on Co followed by insertion into nearby vacancy) emerges as the fastest with the initial O_2_-adsorption step as the rate-limiting step. The overall rate on this surface is predicted to be 3 orders of magnitude faster than on the SrO surface (see Fig. 4 mechanism A9 A20 A23 A26), the latter being reasonably in agreement with the experimental data. Refer to the text for the description and SI for numerical details

**Fig. 6:**
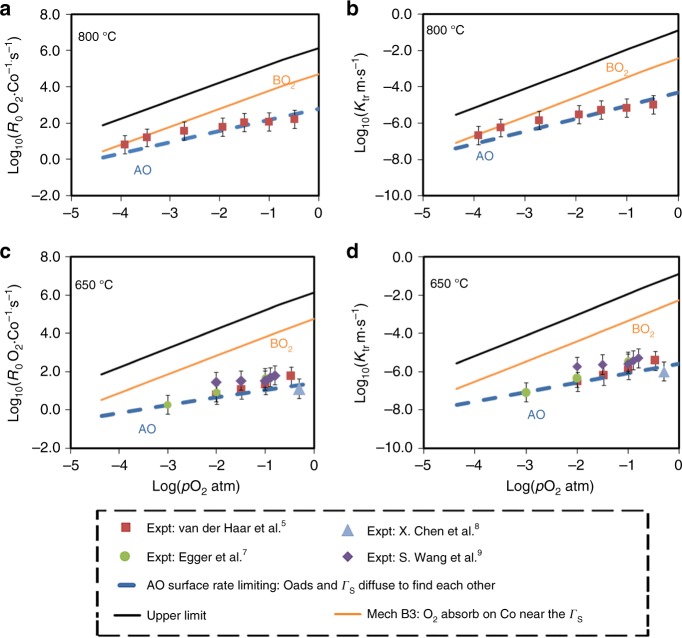
Oxygen partial pressure (*p*O_*2*_) dependence of oxygen exchange rate. Log-log plot of the *p*O_*2*_-dependence of exchange rates (*R*_0_) from various fastest mechanisms on CoO_2_ (Mechanism B3) and SrO terminations (Mechanism A9) of La_0.5_Sr_0.5_CoO_3_ (LSC-50) at 800 °C (**a**) and 650 °C (**b**). Magenta squares, black circles, purple diamonds, and blue triangles refer to experimental exchange rate (log_10_(*R*_0_)) data from Van der Haar et al.^5^, Egger et al.^7^, X. Chen et al.^8^ and extrapolated data from S. Wang et al.^9^ to lower temperature, respectively. Equation (4), along with non-stoichiometry data from Mizusaki et al.^41^ is used to convert the experimental exchange coefficients data (*K*_tr_) to the values of exchange rates (*R*_0_). Rates of key elementary steps of Mechanism B3 for CoO_2_ surface, and AO surface (rate-limiting V_O_ and O_ads_ diffusion) are shown in yellow and dashed-blue respectively. Oxygen adsorption limit is shown as black line. **c**, **d** Corresponding plot of the *p*O_*2*_-dependence of exchange coefficients (*K*_tr_, m·s^−1^) for LSC-50 at 800 °C (**a**) and 650 °C (**b**). Refer to “Methods” section for the details of the fundamental upper-limit for *K*_tr_

The predicted exchange rates *R*_0_ (given in this work in number of O_2_ · Co^−1^·s^−1^, where Co are top or second layer depending on the surface termination) of the fastest oxygen exchange mechanisms are shown in Fig. 4 (SrO surface) and Fig. 5 (CoO_2_ surface) at relevant SOFC conditions (*p*O_*2*_ *=* 0.2 atm, *T* = 650 °C). The calculation details are in the Methods section. Mechanisms A9, A20, A23, A26, and B3 are predicted to be the fastest, and therefore dominant, on the SrO and CoO_2_ surfaces, respectively. As shown in Fig. 4, mechanisms A9, A20, A23, A26 on the SrO termination have one common rate-limiting step, which involves O_ads_ and vacancy lateral diffusion to find each other, with a rate governed by the expression *R*_0_(diffusion) = (8 × *D*_V_ × 2*Γ*_s_ × *Γ*_Oads_)/*d*^2^. This process involves dilute species, so can be modeled accurately in terms of each species undergoing long-range diffusion and undergoing collisions at a rate governed by mean-field theory (see Supplementary Note 2). As illustrated in Fig. 5, mechanism B3 on CoO_2_ termination is limited by the initial O_2_ adsorption and incorporation step (see “Methods” section below for details on calculation of this rate). The thermodynamic stability of the SrO surface suggests that Mechanism A9, A20, A23, A26 should give the best match for experiments, and the result is very close to experimental surface exchange data at *p*O_*2*_ *=* 0.2 atm, *T* = 650 °C^5,7–9^ (blue line of Fig. 4) and over a range of T-*p*O_*2*_ (discussed below and in Fig. 6). It should be noted that the predicted log(*R*_0_) vs. log(*p*O_2_) is very linear, similar to the experimental trends at 650 °C, but missing an apparent curvature observed at 800 °C in Egger’s data and identified by Adler, et al.^11^ as illustrating a quadratic dependence of *R*_0_ on bulk vacancy concentration with changing *p*O_2_. The model trends with *p*O_2_ are governed by bulk oxygen vacancy ***x***_***v***_ and surface oxygen concentration ${\boldsymbol{\Gamma }}_{{\mathrm{sO}}_2}$, which decrease and increase almost linearly with increasing *p*O_2_ in our model, respectively. The ***x***_***v***_ behavior is taken directly from experimental measurement^36^, and therefore we assume it is correct. The *Γ*_Oads_ behavior is expected from simple thermodynamic arguments if O adatoms are thermodynamically ideal, and they are in our model. However, it is possible that the model has missed some electronic transfer to the O adatom, which would weaken binding as the system is oxidized at higher *p*O_2_ and lead to curvature in the log(*R*_0_) vs. log(*p*O_2_) plot. Such a mechanism might be expected to be suppressed at high Sr content as the oxidized system would have reduced electron transfer to the adatom, which is consistent with what is seen in Egger’s data^7^.

The different behavior of these surfaces can be understood in terms of their oxidation state. We focus on *p*O_*2*_ *=* 0.2 atm, *T* = 650 °C but the qualitative arguments given here are true for a wide range of relevant conditions. The SrO surface is more reduced (less oxidized) than the CoO_2_ surface^17^, as the former has balanced nominal charges of [Sr^2+^O^2−^] while the latter is oxidized, with nominal charges [Co^3+^(O^2−^)_2_]. The different oxidation level leads to more stable oxidizing defects (e.g., O adatoms) and less stable reducing defects (e.g., oxygen vacancies) on the SrO vs. CoO_2_ surface. This effect can also be understood as due to compensating polarity of the surface^20^. The differences in the surfaces lead to a dramatic difference in their surface oxygen vacancy concentrations, *Γ*_s_(SrO_surf_) = 6 × 10^−5^ and *Γ*_s_(CoO_2surf_) = 0.28, which difference leads to the vacancy assisted O_2_ dissociation mechanism being much more active on the latter. The 10^2^–10^3^ times faster oxygen exchange rate of CoO_2_ surface vs. SrO surface is due to the high vacancy concentration on CoO_2_, which assists O_2_ adsorption on CoO_2_ through a strong stabilization of O_2_ adsorbate with nearby vacancies. Also, vacancies assist O_2_ dissociation to have a much lower barrier on the CoO_2_ surface than SrO surface.

The importance of surface segregated Sr can now also be understood in terms of surface oxidation state and the active mechanisms. The fastest mechanism (A9, A20, A23, A26) is rate limited by O_ads_ and O_v_ diffusion, and the rate of this step scales with the vacancy content on the SrO surface (Fig. 2a). As the oxygen vacancy content is greatly increased by Sr (*Γ*_s_(LaO_surf_) = 10^−16^ is 10^11^ times smaller than *Γ*_s_(SrO_surf_)), it is clear that Sr enrichment within the AO surface enhances activity by stabilizing surface vacancies for oxygen diffusion and incorporation. This result suggests that for the equilibrated LSC material the Sr both in the bulk and segregated to the perovskite surface is critical for good performance. However, precipitation of Sr compounds (such as oxides, hydroxides and/or carbonates) on the surface, perhaps driven in part by Sr segregation, could potentially degrade performance^22^. Furthermore, overall, AO surface formation is expected to reduce activity vs. the CoO_2_ surface.

These results suggest that LSC-50, and potentially many other perovskite systems, could be greatly enhanced if a metastable BO_2_ termination could be maintained. From a number of recent experiments, the formation of Sr-enriched phase on the surface is known to be concurrent with ORR performance degradation for various perovskite SOFC cathodes^22,25,30,33,37–39^. Crystalline LSC-64 thin-films are known to degrade by one to two orders of magnitude^24,30,33^ in their ORR performance within tens of hours^24^ of preparation or annealing, and to regain performance with etching^33^. Studies of carefully controlled surfaces and Sr and Co deposition have also suggested that Co sites are much more active that Sr^34^. Based on our results above we propose that these changes can be explained as due to the metastable and highly active CoO_2_ surface being present in as-received or etched materials and being rapidly replaced by a Sr rich AO surface as the system equilibrates at high temperature. The appearance of degradation at temperatures around 500–600 °C^22,30^ and its time scale of tens of hours^24^ are both consistent with the temperature and time scales associated with the cation mobility required for moving La and Sr to the surface^40^. Many authors have suggested the Sr itself is driving the degradation^22,24,30^, although our model suggests that it is the formation of the AO surface and not Sr itself that leads to the degradation, at least before the formation of Sr-based precipitates on the surface. We note that our proposed mechanism for the degradation is quite speculative and that other mechanisms, e.g., the formation of secondary Sr rich phases and site poisoning, might play a significant or even dominant role. Further work on LSC and related systems is needed to validate these speculations.

Table 1 lists the DFT-predicted reaction kinetic barriers, overall T-*p*O_2_ dependences, and reaction rates (*R*_0_) at SOFC conditions. Figure 6a, b show the predicted T-*p*O_2_ dependence of *R*_0_ of the key mechanisms of LSC-50. Figure 6c, d show the corresponding plots for *K*_tr_. The *p*O_2_ dependence of *R*_0_ or *K*_tr_ is a function of the kinetic symmetry parameter (*β*), which is not readily determined from the DFT calculations, and hence value *β* is fit to the experimental *p*O_2_ dependence. The experimental *R*_0_ is derived from the reported *K*_tr_ data^5,7^, Eq. (4) in the “Methods” section, and known defect chemistry^41^. Only the fastest mechanisms on both surfaces (BO_2_, orange, and AO, blue dashed line) are shown for clarity, along with the initial O_2_ adsorption rate (black). The predicted slope of log_10_(*R*_0_) vs. log_10_(*p*O_*2*_) for SrO termination is 0.4–0.6 in Fig. 6, matching well with the range 0.4–0.8 of slope from these experiments in Table 1. The Arrhenius activation energy (describing T-dependence) of experimental *K*_tr_ for LSC-50^5,7^ (and the closely related La_60_Sr_40_CoO_3−δ_^7^) ranges from 0.99–1.57 eV for 0.1 atm *p*O_*2*_ and 0.55–1.67 eV for 10^−3^ atm *p*O_*2*_. The narrow range of our predicted activation energies for the SrO surface of 1.51 eV (for 0.2 atm *p*O_*2*_) and 1.31 eV (10^−3^ atm *p*O_*2*_), respectively (AO surface) agree well with the upper range of these values, although more experimental data is clearly needed for quantitative comparison (see Table 1). Within the uncertainties in the experiments and ab initio energetics, our results on model SrO termination agree very well with the experimental T-*p*O_2_ dependence, providing validation for the model. The agreement between the experiment and the model for Sr-rich AO termination is also fully consistent with the fact that this surface is the thermodynamically stable surface of LSC^22–24,26,31^. Figure 6 also shows that the oxygen exchange rates (*R*_0_) and oxygen exchange rate coefficient (*K*_tr_) at the CoO_2_ surface can be two to three orders of magnitude larger than SrO surface, for wide range of T-*p*O_*2*_.

**Table 1:** Temperature-dependences and kinetic symmetry parameter

	Kinetic symmetry parameter, *β*	Rate-limiting step	Slope of log_10_(*R*_0_) vs. log_10_(*p*O_2_)	Kinetic barrier for *K*_tr_ (eV)	Kinetic barrier for *k*(*T*)^a^ (eV)	log_10_*R*_0_ (#O_2_·Co^−1^·s^−1^) (650 °C, 0.2 atm *p*O_*2*_)
Experiment: van der Haar et al.^5^ (LSC(50%Sr))	–	–	0.6 (650 °C), 0.8 (low-*p*O_2_) and 0.4 (high-*p*O_2_) at 750 °C	0.99 (0.2 atm), 1.67 (1E−03 atm)	0.86 (0.2 atm), 1.49 (1E−03 atm)	1.42
Experiment: Egger et al.^7^ (LSC(50%Sr))	–	–	0.6 (at 750 °C), 0.7 (at 650 °C)	1.57 (0.1 atm), 1.23 (1E−03 atm)	1.23 (0.1 atm), 0.94 (1E−03 atm)	~1.74 (extrapolated to 0.2 atm *p*O_2_)
Experiment: Egger et al.^7^ (LSC(40%Sr))	–	–	0.8 (750 °C), 0.7 (650 °C)	1.48 (0.1 atm), 1.03 (1E−03 atm)	1.05 (0.1 atm), 0.7 (1E−03 atm)	~1.36 (extrapolated to 0.2 atm *p*O_2_)
Experiment: Lu et al. (LSC(40%Sr))	–	–	0.6 (750 °C), 0.4 (650 °C)	1.32^b^ (0.2 atm), 0.55^b^ (1E−03 atm)	0.84^b^ (0.2 atm), 0.22^b^ (1E−03 atm); 0.47 (fitted)^c^	0.35 (at 750 °C)
CoO_2_ termination: Mech. B2	0.8	CoO_2_ss = null (dissociation)	0.5	0.9 (0.2atm), 0.7 (1E−03 atm)	0.68 (0.2 atm), 0.5 (1E−03 atm)	2.2
CoO_2_ termination: Mech. B3	0.5	O_2_ + s + s = sO_2_ + s(far) (insertion into vacancy)	1.0	0.55 (0.2 atm), 0.44 (1E−03 atm)	0.33 (0.2 atm), 0.24 (1E−03 atm)	4.1 (fastest on CoO_2_ layer)
CoO_2_ termination: Mech. B4	0.8	CoO_2_s = CoO+s(far) (dissociation)	0.6	1.74 (0.2 atm), 1.79 (1E−03 atm)	1.53 (0.2 atm), 1.59 (1E−03 atm)	2
SrO termination: Mech. A9 A20 A23 A26	1	*O(O_top_) + s(far) = *O(O_top_) + s(near) (diffusion)	0.4 (650 °C), 0.6 (800 °C)	0.86 (0.2 atm), 0.66(1E−03 atm)	0.64 (0.2 atm), 0.43 (1E−03 atm)	1.1 (fastest on SrO layer)

List of *p*O_2_, T-dependences and choice of the kinetic symmetry parameter (*β*) used in the modeling of ORR mechanisms B2, B3, B4 of CoO_2_ termination and the mechanisms A9 and A20 of the SrO-termination. These mechanisms emerge as amongst the fastest in terms of oxygen exchange rates (#O_2_·Co^−1^·s^−1^). The unit O_2_·Co^−1^·s^−1^ is number of adsorbed oxygen molecular at one surface Co area per second. Temperature and *p*O_2_-dependence of experimental data are calculated using the reported *K*_tr_ data and converting to *R*_0_ data. All values for various ORR mechanisms are computed for LSC(50%Sr). Experimental data for LSC(40%Sr) is given as a reference

^a^Arrhenius dependence of *k*(*T*) is negative slope of the plot of ln(*k*(*T*)) vs. ln(1/(*k*_B_*T*)); where *k*(*T*) is defined after Lu et al. as $R_0 = P_{{\mathrm{O}}_2}^{{\mathrm{gas}}} \times \delta ^2 \times k(T)$

^b^Values calculated using reported *R*_0_ data from Lu et al.^61^

^c^This value is reported in Lu et al. and uses *k*(*T*) as a fit of *R*_0_ vs. *p*O_*2*_* δ*^2^

**Fig. 7:**
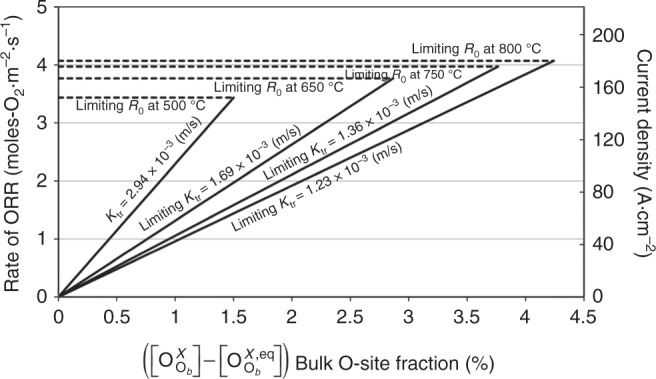
Upper limit surface exchange rate. Oxidation reduction reaction (ORR) rates (moles-O_2_·m^−2^·s^−1^, or equivalently current density in A·cm^−2^) as a function of typical values of ($\left[ {{{\mathrm{O}}}_{{{\mathrm{O}}}_{{b}}}^{{X}}} \right]- \left[ {{{\mathrm{O}}}_{{{\mathrm{O}}}_{{b}}}^{{{X}},{{\mathrm{eq}}}}} \right]$, moles-O·m^−3^) at SOFC operating conditions (0.2 atm, temperatures in the range of 500–750 °C, cell voltage of 1 V). Horizontal dashed lines show limiting ORR rates at particular temperatures and corresponding limiting *K*_tr_ (m·s^−1^) are shown as slanted lines of the type *y* = *mx*, with slope = *K*_tr_

Figure 6 shows the O_2_ adsorption step (black). As the first elementary step for all ORR mechanisms, this step sets an upper fundamental limitation for ORR on any cathode surface. This limit is set by the kinetic theory of gas absorption (expressed as *R*_0_ in Fig. 6a, b and *K*_tr_ in Fig. 6c, d). Details of these estimates are in the “Methods” section and Fig. 7. At relevant SOFC conditions (0.2 atm *p*O_*2*_, 650 °C), the upper-limit exchange rate set by this step corresponds to *R*_0_ ~ 3.63 × 10^5^ O_2_·Co^−1^·s^−1^, *K*_tr_ ~ 1.69 × 10^−3^ m·s^−1^, and an exchange current density of ~170 A·cm^−2^ (taking into account the experimental lattice parameters and 4 e^−^ carried per O_2_ molecule). Thus at these conditions of T-*p*O_*2*_, we predict that these values are an absolute upper-limit achievable for any active SOFC cathode surface. The *K*_tr_ for the highly active, but metastable, surface provided by the CoO_2_ termination of LSC-50 (Fig. 6c, d, orange line) may get close to this upper-limit, which suggests significant opportunity for improving ORR activity for complex oxides. We note that the kinetic model developed here can also be used to predict overpotential as a function of current density. This is done and the results shown to compare very favorably to experiments in “Methods” section, further supporting the validity of the model.

## Discussion

In summary, understanding, controlling, and enhancing oxygen exchange on oxide surfaces is critical to the success of many technologies, especially for lowering the operating temperature of SOFCs. We have developed an ab initio based microkinetic model that provides quantitative predictive ability for oxygen exchange rates vs. temperature and *p*O_*2*_ on La_0.5_Sr_0.5_CoO_3−δ_ (LSC-50). The model identifies surface diffusion and adsorption of O_2_ as the rate-limiting steps on Sr-rich (La,Sr)O-terminated and CoO_2_-terminated surfaces, respectively, and predicts exchange rates on the stable SrO surface in excellent agreement with experiments. The O_2_ reduction on the metastable CoO_2_ surface is predicted to be orders of magnitude faster than on the thermodynamically stable SrO surface, suggesting that the loss of CoO_2_ termination is most likely the reason for the widely-observed degradation of oxygen exchange rates over tens of hours of annealing. Also, while the (La,Sr)O surface is generally predicted to be less active than the CoO_2_ surface, the segregation of Sr to form a SrO termination is shown to be critical to good performance on this termination. Finally, this work estimates a fundamental upper-limit of oxygen exchange rate set by the O_2_ adsorption step. The metastable, pure CoO_2_ surface of LSC-50 is predicted close to this limit under relevant SOFC conditions, enabling a 2–3 orders of magnitude faster oxygen exchange than Sr rich AO surface. This result demonstrates an inherent potential for enormously enhanced performance in this and related materials if a high concentration of the transition metals can be permanently stabilized on the surface.

## Methods

### Modeling approach

Calculations were performed with the Vienna Ab initio Simulation Package^42,43^ (VASP) using Density Functional Theory (DFT) and the Projector-Augmented plane-Wave (PAW)^44^ method. Exchange-correlation was treated in the Perdew-Wang-91 (PW-91^45^) Generalized Gradient Approximation (GGA) with electronic configurations of La (5s^2^ 5p^6^ 6s^2^ 5d^1^), O_*s* (‘soft’ oxygen pseudopotential, 2s^2^ 2p^4^), Sr (4s^2^ 4p^6^ 5s^2^) and Co (3d^8^ 4s^1^). In order to take into account the effect of the strongly correlated electronic systems we applied the rotationally invariant GGA+U^46^ method, with *U* and *J* entering as an effective parameter, *U*_eff_ *=* 5.6 eV, which was fit so our bulk oxygen vacancy formation energy matches the experiment data from Mizusaki et al.^41^. We used the U-ramping method^47^ to search the global minimal energy. Bulk calculations were simulated with 80-atom unit cell (2*a*_pc_ × 2*a*_pc_ × 4*a*_pc_); *a*_pc_ is the pseudo-cubic lattice constant of LSC). In calculating the bulk oxygen vacancy formation energy, the oxygen vacancy was placed such that two of its nearest neighbor A-sites are filled with doped Sr and two with La. The surface calculations were done with an eight-layer slab (2_ac_ × 2_ac_ × 4_ac_ with 4 layers of AO, 4 layers of BO_2_). Slabs were asymmetric (had both AO and BO_2_ terminations) and dipole corrections^48,49^ were used. In the calculations of the energies of adsorbed oxygen (*O, *O_2_) on either (001) surfaces, we embedded a vacancy on the exposed CoO_2_ termination to represent the significant vacancy concentration expected on this surface. In case of adsorbates on the CoO_2_ termination itself, the adsorbate and surface vacancy were separated by more than 5 Å in order to reduce the effects of neighbor interactions, except in the case while treating adsorbate with a nearest neighbor vacancy itself. To study the ORR on Sr-rich AO-termination, SrO-terminated surfaces were created with a 2 × 2 × 4 or 80-atom supercell similar to the CoO_2_ slabs above, but keeping a 100% Sr concentration at the top surface. The total concentration of Sr in the slab was maintained at 50% Sr. This cell represents a model Sr-enriched LSC surface that is expected to be largely covered with an SrO-termination^22,24^. All energies are free energies with vibration contributions included for gaseous, lattice and surface atoms. For the details of types of ORR intermediates, and choice of thermodynamic reference states, reader is referred to the Supplementary Note 1. The energy cutoff was set to 450 eV and the brillouin zone was sampled by a Monkhorst-Pack k-point mesh of (2 × 2 × 1) for a 2 × 2 × 4 perovskite supercell. The 3d-electron configuration of Co(III) was modeled with Intermediate Spin (IS) state $\left( {t_{2g}^5e_g^1} \right)$. Using GGA+U, the intermediate spin state in LSC was found to be the most stable and to yield the most accurate oxygen vacancy formation energies^50^ among the different spins (low, intermediate, high spin), hence all the spin-polarized calculations were relaxed from an IS state^51^. The magnetic configuration for electron spins was chosen to be Ferromagnetic (FM) after the work of Lee et al.^17^. Use of Ferromagnetic (FM) configuration and use of pseudocubic lattice is justified as it closely simulates the high-Temperature nature of LSC lattice^17^. The bulk and slab energies were converged to within 3 meV per formula unit with respect to the k-points and energy cutoff. Structural relaxations were converged to within 1 meV per atom. Surface structures were calculated by truncating the bulk with insertion of 10 Å vacuum above the surface under periodic conditions.

Surface exchange rates are determined by enumerating possible pathways (mechanisms) and finding the rates of each elementary step, setting the total rate to that slowest elementary step in each mechanism. For the surfaces of LSC-50, we enumerate and organize different mechanistic pathways of ORR in terms of their adsorption and dissociation sites. Supplementary Tables 2 and 5 show the list mechanisms organized according to the adsorption (rows) and dissociation sites (columns) of the (La,Sr)-O and CoO_2_ termination, respectively. A typical (La,Sr)-O termination without a specific ordering of the A-site cations would have (*m*=)14 probable sites for oxygen adsorption, and (*n*=) 14 sites for dissociation. Only a few of the probable *m* × *n* site combinations give rise to molecular pathways that are meaningful and unique. In case of (La,Sr)-O and CoO_2_ terminations respectively, we identify 41 and 12 unique molecular pathways that can describe the site-specific adsorption and dissociation steps of ORR. Refer to Supplementary Tables 3, 6 and 7 for the details of molecular pathways, Supplementary Tables 1 and 4 for neutral building units and their chemical potentials, and Fig. 3 for the reaction energy landscape.

### Rate expressions

In the following, we formulate the rate equations for individual elementary steps of any oxygen exchange mechanism. We use rate-limiting step approximation to then calculate the rate of oxygen exchange mechanisms. In other words, given the rates of all elementary steps of a specific oxygen exchange mechanism, the rate of oxygen exchange through this mechanism will be the slowest of the series of elementary steps and the rate of overall ORR, the sum of all parallel mechanisms, dominated by the fastest ORR mechanism. Applying transition state theory, the net kinetic rate for an elementary step, i of a reaction, viz. *X* + *Y* = *Z* can be written as^11^,

2 $${{r}} = {{k}}_{{\mathrm{i}}}{{{\boldsymbol{\Gamma}} }}_{{X}}{{{\boldsymbol{\Gamma}} }}_Y{\mathrm{e}}^{ - {\mathrm{\Delta }}{{G}}_{{{\mathrm{f}}},{{\mathrm{i}}}}^0/({{k}}_{{\mathrm{B}}}{{T}})}{\mathrm{e}}^{ - (1 - {{\beta }}){\mathrm{\Delta }}{{E}}_{{\mathrm{i}}}^0/({{k}}_{{\mathrm{B}}}{{T}})} \times \left[ {1 - {\mathrm{e}}^{ - {\mathrm{\Lambda }}_{{\mathrm{i}}}/({{\lambda }}_{{\mathrm{i}}}{{k}}_{{\mathrm{B}}}{{T}})}} \right]$$

where *k*_i_ is a temperature-independent pre-exponential factor, Δ*G*_f,i_ is the unperturbed free-energy barrier for the reaction step (evaluated at Δ*E*_i_ = 0), *Γ*_*X*_ and *Γ*_*Y*_ are the concentrations of reactants *X* and *Y*, *β* is a reaction symmetry parameter analogous to that used in electrochemical kinetics, *Λ*_i_ is the total free-energy driving force for the reaction step, *λ*_i_ is the stoichiometric coefficient of the elementary step, and Δ*E*_i_ is the energy-shift component of the thermodynamic driving force for the i^th^ elementary step. Equation (15) gives the net forward rate (*r*_i_) of a single elementary step(i) of any reaction mechanism. The driving force for step i, *Λ*_i_ is the difference in the free energy of reactants minus products. The driving force for overall oxygen incorporation is the deviation in the chemical potential of O_2_ due to a deviation in its external pressure from equilibrium. Under the rate-limiting step approximation, the driving force (*Λ*) for the total reaction is assumed to be contributed in full by the rate-limiting reaction step, i.e., (*Λ*_i_), or, ${{{\boldsymbol{\Lambda}} }}_{\mathrm{i}} = {{\boldsymbol{\Lambda}}/}\lambda _{\mathrm{i}} = k_{\mathrm{B}}T\lambda _{\mathrm{i}}^{ - 1}{\mathrm{ln}}\left( {P_{{{\mathrm{O}}_2}}^{\mathrm{gas}}{\mathrm{/}}f_{{{\mathrm{O}}_2}}^{\mathrm{gas}}} \right)$, where *λ*_i_ is the stoichiometric ratio between reaction i and overall reaction under quasi-equilibrium conditions, $P_{{{\mathrm{O}}_2}}^{\mathrm{gas}}$ is the partial pressure of oxygen in the gas relative to atmospheric pressure and, $f_{{{\mathrm{O}}_2}}^{\mathrm{gas}}$ the oxygen fugacity in the oxide. At no net driving force (*Λ* = 0), the forward and backward rates will be identical, represented by an equilibrium exchange rate, *R*_0_. Net rate can be written in terms of *R*_0_ as,

3a $${{r}} = {{R}}_0[1 - {\mathrm{e}}^{ - { {{\boldsymbol{\Lambda}} }}_{{i}}/({{k}}_{\mathrm{B}}{{T}})}],\quad {\mathrm{where,}}$$

3b $$R_0 = k_{\mathrm{i}}{{\boldsymbol{\Gamma}}}_X{{\boldsymbol{\Gamma}}}_Y{\mathrm{e}}^{ - {\mathrm{\Delta }}G_{\mathrm{f,i}}^0/(k_{\mathrm{B}}T)}{\mathrm{e}}^{ - (1 - \beta ){\mathrm{\Delta }}E_{\mathrm{i}}^0/(k_{\mathrm{B}}T)}$$

The ‘chemical’ surface exchange rate coefficient *K*_tr_ can further be calculated in terms of *R*_0_, assuming a linearity in Λ-dependence of total rate *r* (see Adler et al.^11^ page 98),

4 $${{K}}_{\mathrm{tr}} = - \frac{{2{{R}}_0}}{{{{x}}_{{\mathrm{v}}}{{C}}_0{{\gamma }}}}$$

Where *x*_v_ is bulk oxygen vacancy site fraction (unitless), $\gamma = \left( {\partial {\mathrm{ln}}x_{\mathrm{v}}{\mathrm{/}}\partial {\mathrm{ln}}f_{{{\mathrm{O}}_2}}^{\mathrm{gas}}} \right)_T$ is a material specific thermodynamic factor (unitless) and *C*_0_ (in moles of oxygen lattice sites·m^−3^) is the oxygen site concentration in the bulk. For convenience, we stick to the discussion of *R*_0_ in the units of (#O_2_·Co^−1^·s^−1^), although the results can be translated to the exchange rate coefficient (*K*_tr_, m·s^−1^) using Eq. (4) and appropriate unit conversions. Supplementary Table 8 lists all the key thermodynamic and kinetic parameters used in this work, and gives the parameter value, or refers to the specific tables and figures where to find the information within “Methods” section and main text.

### DFT corrections

Corrections were applied to DFT energies of surface adsorbates and kinetic barriers, as explained below:

Identifying electron doping contributions: When dealing with formation and adsorption energies, it is useful to separate out the energetics of the process that is associated with electron donation/removal from the Fermi level, as this quantity depends on the overall defect chemistry and therefore *T* and *p*O_2_. In this work, the DFT simulations of bulk and surfaces of LSC-50 were performed with 2 × 2 × 4 supercells, or with 16 formula units (FUs) of LSC-50, which leads to significant electron donation/removal contributions in the basic DFT energies. For example, when simulating vacancy formation energy, 1 oxygen vacancy is simulated in such a 2 × 2 × 4 supercell, which dopes 2 electrons into the supercell. Following the rigid band formalism for LSC-50 from Lankhorst et al.^52^, the two electrons will be doped at the Fermi level, and the Fermi level will be incremented by $\frac{n}{{16 \times {g}(E_{\mathrm{F}})}}$, where *n* is doping concentration (per 16 FU supercell), *g*(*E*_F_) is the density of states at the Fermi level. This density of states has the value *g*(*E*_F_) = 4/*a* = 1.71 eV^−1^ FU^−1^, where the parameter *a* = 2.34 eV is the vacancy interaction parameter from Mizusaki et al.^41^. Since we calculate oxygen vacancy formation energy as a difference between a supercell with one vacancy and a perfect (no vacancy) one, an electron donation correction energy of $E_{\mathrm{DFT}}^{\mathrm{don}} = \int_{n = 0}^{n = 2} \frac{n}{{16 \times {g}(E_{\mathrm{F}})}}\mathrm{d}n$ or $\frac{2}{{16 \times{g}(E_{\mathrm{F}})}}$ needs to be removed (subtracted) from the vacancy formation energy to remove this energy contribution. Similarly when calculating an adsorption energy of species *O_2_^1−^, an energy of $E_{\mathrm{DFT}}^{\mathrm{don}} = \int_{n = 0}^{n = 1} \frac{n}{{16 \times {g}(E_{\mathrm{F}})}}\mathrm{d}n = \frac{n}{{32 \times {g}(E_{\mathrm{F}})}}$ needs to be added to the adsorption energy. These corrections are identified by a symbol $E_{\mathrm{DFT}}^{\mathrm{don}}$ in Eqs. (6) and (8) below.

Correction to adsorbate energy that contains O–O bond: As the GGA calculations overestimate the binding energy of oxygen molecule, a standard O_2_-overbinding correction (0.33 eV/O2 from the work of Lee et al.^17^) is applied for the DFT-energy of O_2_, and surface adsorbates that retain an O–O bond (species of the type *O_2_^0.5−^, *O_2_^1−^, *O_2_^2−^, where ‘*’ denotes an adsorbate).

Correction to kinetic barrier: The energy component of the driving force shifts the reaction barrier by (1 − *β*)Δ*E*_i_, giving an effective reaction barrier ${\mathrm{\Delta }}G_{\mathrm{eff,i}} = {\mathrm{\Delta }}G_{\mathrm{f,i}}^0 - (1 - \beta ){\mathrm{\Delta }}E_{\mathrm{i}}$, where *β* is empirical symmetry parameter (usually ranges from 0.5 to 1). For a reaction step involving the breaking of oxygen dimmer bond (O–O dissociation step), applying the above DFT oxygen overbinding correction of +0.33 eV on the initial state of this reaction would result in increasing the driving force for forward reaction, hence decreasing the effective reaction barrier. For example, the corrected effective barrier for oxygen dissociation at divacancy of Mechanism B2 (Step 2 of Mechanism B2), is $\Delta G_{\mathrm{eff,i}} = {\mathrm{\Delta }}G_{\mathrm{f,i}}^0 - \left( {1 - \beta } \right){\mathrm{\Delta }}E + (1 - \beta ) \times 0.33$.

Besides these, errors remain in any DFT calculated reaction energies and reaction barriers. These errors are discussed in detail in Supplementary Note 2, where we estimate an error bar of ±1.5 order of magnitude.

### Determining vibrational free energy of solid phase oxygen

In this work we use thermodynamic parameters for bulk oxygen from the work of Mizusaki et al.^41^. In particular the chemical potential of LSC-50 bulk oxygen in reference to O_2,gas_ is set using their reported parameters ${\mathrm{\Delta }}h_{\mathrm{O}}^0$, Δ*S*_0_ and *a* (Figure 13a of Mizusaki et al.^41^). However, we need to make use of bulk defect energies from DFT and vibrational models relative to the oxygen gas (obtained from an empirical model from NIST^53^). To assure that our DFT-based model matches the Mizusaki model here we fit an enthalpy correction and Einstein temperature for our DFT-based model. The fit matches the free energy change of bulk vacancy formation at relevant SOFC conditions between the our calculated and Mizusaki models. This approach is illustrated as follows:

Reaction: oxygen from bulk LSC goes to oxygen (1/2 O_2_) in gas forming a bulk vacancy,

5 $${{\mathrm{O}}}_{{\mathrm{b}}}^{{X}} \to \mathrm{V}_{{{\mathrm{O}}}_{{\mathrm{b}}}}^{ \cdot\cdot } + 2{{\mathrm{e}}}^ - + {\mathrm{1/2}}\,{{\mathrm{O}}}_2;{\mathrm{\Delta }}{{G}}_1 = {\mathrm{1/2}}\,{{G}}_{{{\mathrm{O}}}_2} - {{G}}_{{{\mathrm{O}}}_{\mathrm{b}}}$$

Δ*G*_1_ represents an ideal, non-configurational Gibb’s free energy change of the formation of oxygen vacancy. Δ*G*_1_ can be expanded as sum of DFT and T-dependent terms and a constant correction term,

6 $$\begin{array}{*{20}{l}} {{\mathrm{\Delta }}{{G}}_1} \hfill & = \hfill & {\left( {{\mathrm{1}}{\mathrm{/}}{\mathrm{2}}\left( {{{E}}_{{{\mathrm{O}}}_2}^{{{\mathrm{DFT}}}} + {\mathrm{\Delta }}{{h}}_{{\mathrm{O}}}^0 + 2{{H}}_{{{\mathrm{vib}}},{{\mathrm{O}}} - {{\mathrm{solid}}}}^{{{T}} = {{T}}^0}} \right)} \right.} \hfill \\ {} \hfill & {} \hfill & {\left. { - \left( {{{E}}_{{{n}}_{{\mathrm{b}}}}^{{{\mathrm{DFT}}}} - {{E}}_{{{n}} - 1_{{\mathrm{b}}}}^{{{\mathrm{DFT}}}}} \right) - {{E}}_{{{\mathrm{DFT}}}}^{{{\mathrm{don}}}}} \right)} \hfill \\ {} \hfill & {} \hfill & { + {\mathrm{1}}{\mathrm{/}}{\mathrm{2}}\left( {{{H}}_{{{\mathrm{O}}}_2}^{{{\mathrm{NIST}}}}\left( {{T}} \right) - {{H}}_{{{\mathrm{O}}}_2}^{{{\mathrm{NIST}}}}\left( {{{T}}^0} \right) - {{TS}}_{{{\mathrm{O}}}_2}^{{{\mathrm{NIST}}}}} \right)} \hfill \\ {} \hfill & {} \hfill & { - \mathop {\sum }\limits_{{{i}} = 1}^3 {\kern 1pt} {{k}}_{{\mathrm{B}}}{{T}}{\mathrm{ln}}\left( {2{\mathrm{sinh}}\left( {{{\theta }}_{{{E}},{{i}}}{\mathrm{/}}2{{T}}} \right)} \right) - {\mathrm{\Delta }}{{h}}_{{\mathrm{O}}}^{{{\mathrm{corr}}}}} \hfill \end{array}$$

**Fig. 8:**
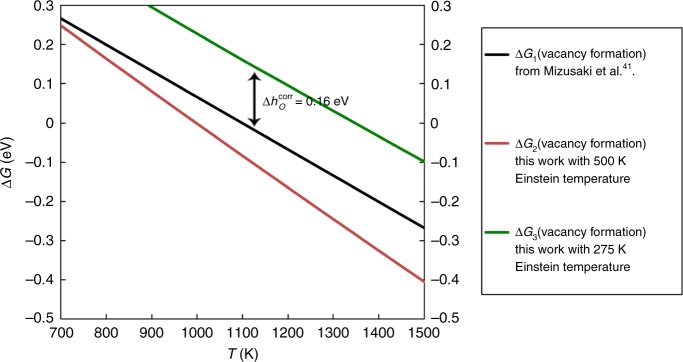
La_0.5_Sr_0.5_CoO_3_ (LSC-50) non-configurational formation free energy. Comparison of ideal, non-configurational vacancy formation free energy for LSC-50 from Mizusaki et al.^41^ (= −1 × Δ*G*^0^ = −Δ*h*^0^ + *T* × Δ*S*^0^, black line) to the ideal, non-configurational vacancy formation free energy calculated in this work (Δ*G*_1_, using DFT and NIST database^53^, according to Eq. (6). Details of models for Δ*G*_1_: 3-vibrational modes of oxygen in the LSC (50%Sr) solid are treated with Einstein temperatures of 275 K (blue line), 500 K (magenta line). Choice of Einstein temperature of 275 K (blue line) matches the Temperature-slope of Δ*G*_1_ to the slope of −1 × Δ*G*^0^ from Mizusaki et al. (black-line) with a constant deviation of +0.16 eV, which we denote as Δ*h*_O_^corr^ in this work

Where, $E_{\mathrm{O}_2}^{\mathrm{DFT}}$ is the DFT-GGA(PW-91^45^) predicted energy of isolated O_2_ molecule (=−9.09 eV/O_2_^17^), term ${\mathrm{\Delta }}h_{\mathrm{O}}^0$ is the O–O overbinding correction added to the O_2_ DFT energy. Term ${\mathrm{\Delta }}h_{\mathrm{O}}^{\mathrm{corr}}$ (=0.33 eV/O_2_ from Lee et al.^17^) is the O–O overbinding relative to lattice oxygen at standard conditions (*T*^0^ = 298.15 K), hence a term $2H_{\mathrm{vib,O - solid}}^{T = T^0}$ (vibrational enthalpy of two lattice-O at *T*^0^ = 298.15 K) is added to avoid double-counting. Term $E_{n_{\mathrm{b}}}^{\mathrm{DFT}}$ is the DFT energy of a bulk supercell with *n* atoms, $E_{n - 1_{\mathrm{b}}}^{\mathrm{DFT}}$ is the DFT energy of a bulk supercell with 1 oxygen vacancy, $E_{\mathrm{DFT}}^{\mathrm{don}}$ is the electron donation correction energy (explained above), $H_{\mathrm{O}_2}^{\mathrm{NIST}}$ and $S_{\mathrm{O}_2}^{\mathrm{NIST}}$ are the enthalpy (relative to 298.15 K) and entropy (absolute scale) of oxygen gas, from NIST database^53^, ${\mathrm{\Delta }}h_{\mathrm{O}}^{\mathrm{corr}}$ is correction term, which is explained as follows. We have used Einstein model to treat vibrational free energy of lattice-oxygen, with *θ*_E_ as the effective Einstein temperature. A similar expression for the ideal, non-configurational free energy of bulk oxygen vacancy formation can be written based on parameters from Mizusaki et al.^41^, viz. −1 × Δ*G*^0^ = Δ*h*^0^ − *T* × Δ*S*^0^, where both Δ*h*^0^ and Δ*S*^0^ are enthalpy and entropy of bulk lattice oxygen (relative to gas-O with an arbitrary reference) referred from Figure 13a of Mizusaki et al.^41^, and the negative sign accounts for the vacancy formation instead of bulk-O formation. As shown in Fig. 8 below, tuning Δ*G*_1_ with *θ*_E_ = 275 K matches the slope (relative to temperature) of −1 × Δ*G*^0^. Any other approximations to the Einstein temperature (such as *θ*_E_ = 500 K shown as example), will set Δ*G*_1_ and −1 × Δ*G*^0^ on different slopes, in other words would give a wrong estimation of the relative entropy of oxygen.

The quantity Δ*G*_1_ when calculated with Einstein temperature of 275 K and corrected with ${\mathrm{\Delta }}h_{\mathrm{O}}^{\mathrm{corr}}$ (=+0.16 eV), then equals −1 × Δ*G*^0^ from Mizusaki et al.^41^ and sets the bulk vacancy site fraction (*x*_v,bulk_) at given T-*p*O_*2*_:

7 $${\mathrm{\Delta }}{{G}}_1 + 2{{E}}_{{\mathrm{F}}}\left( {{{x}}_{{{\mathrm{v}}},{{\mathrm{bulk}}}}} \right) = - {{k}}_{{\mathrm{B}}}{{T}}{\mathrm{ln}}\left( {\frac{{{{x}}_{{{\mathrm{v}}},{{\mathrm{bulk}}}} \cdot \left( {{{P}}_{{{\mathrm{O}}}_2}^{{{\mathrm{gas}}}}} \right)^{1/2}}}{{1 - {{x}}_{{{\mathrm{v}}},{{\mathrm{bulk}}}}}}} \right)$$

2*E*_F_ term accounts for the increment in the Fermi level^52,54^ of LSC-50 due to 2 electrons added to the Fermi level, when a vacancy is created. *E*_F_ = 1.5 × *a* × *x*_v,bulk_^11^, where parameter *a* is referred from Mizusaki et al.^41^. In all the calculations of chemical potential of surface oxygen species (adsorbates, surface vacancy etc.), the bulk-like modes of surface oxygen are treated with Einstein model with an Einstein temperature of 275 K.

### Determining surface oxygen energetics by referencing to bulk

To minimize possible DFT errors we have taken the energies of all oxygen adsorbates and surface vacancies relative to the bulk model. As the bulk model is fit to experiments, this allows surface O energetics to be controlled by just the relative energies of surface and bulk O, which we expect to lead to significant cancellation of errors.

Consider first the example of formation of a surface vacancy,

$$\mathrm{O}_{\mathrm{s}}^X \to \mathrm{V}_{\mathrm{O}_{\mathrm{s}}}^{ \cdot\cdot} + 2\mathrm{e}^ - + 1{\mathrm{/}}2\,\mathrm{O}_2;{\mathrm{\Delta }}G_2 = 1{\mathrm{/}}2\,G_{\mathrm{O}_2} - G_{\mathrm{O}_{\mathrm{s}}}$$

8 $$\begin{array}{*{20}{l}} {\Delta {{G}}_2} \hfill & = \hfill & {\left( {1{\mathrm{/}}2\left( {{{E}}_{{{\mathrm{O}}}_2}^{{{\mathrm{DFT}}}} + \Delta {{h}}_{{\mathrm{O}}}^0 + 2{{H}}_{{{\mathrm{vib}}},{{\mathrm{O}}} - {{\mathrm{solid}}}}^{{{T}} = {{T}}^0}} \right)} \right.} \hfill \\ {} \hfill & {} \hfill & {\left. { - \left( {{{E}}_{{{n}}_{{\mathrm{s}}}}^{{{\mathrm{DFT}}}} - {{E}}_{{{n}} - 1_{{\mathrm{s}}}}^{{{\mathrm{DFT}}}}} \right) - {{E}}_{{{\mathrm{DFT}}}}^{{{\mathrm{don}}}}} \right)} \hfill \\ {} \hfill & {} \hfill & { + 1{\mathrm{/}}2\left( {{{H}}_{{{\mathrm{O}}}_2}^{{{\mathrm{NIST}}}}\left( {{T}} \right) - {{H}}_{{{\mathrm{O}}}_2}^{{{\mathrm{NIST}}}}\left( {{{T}}^0} \right) - {{TS}}_{{{\mathrm{O}}}_2}^{{{\mathrm{NIST}}}}} \right)} \hfill \\ {} \hfill & {} \hfill & { - \mathop {\sum }\limits_{{{i}} = 1}^3 {{k}}_{{\mathrm{B}}}{{T}}{\mathrm{ln}}\left( {2{\mathrm{sinh}}\left( {{{\theta }}_{{{\mathrm{E}}},{{i}}}{\mathrm{/}}2{{T}}} \right)} \right)} \hfill \\ {} \hfill & {} \hfill & { - \Delta {{h}}_{{\mathrm{O}}}^{{{\mathrm{corr}}}}} \hfill \end{array}$$

We assume that the modes of vibration of the surface-lattice-O are identical to the modes of the bulk-O. Two electrons are doped at the Fermi level in 2 × 2 × 4 simulation supercells for both surface and bulk DFT calculations, implying the terms ${{E}}_{{{\mathrm{DFT}}}}^{{{\mathrm{don}}}}$ (see section on DFT corrections above) in both Δ*G*_1_ and Δ*G*_2_ will be equal. Hence, comparing the expressions (6) and (8), difference between Δ*G*_1_ and Δ*G*_2_ is depends on only the difference in the DFT terms:

9 $$\begin{array}{*{20}{l}} {{\mathrm{\Delta }}{{G}}_2 - {\mathrm{\Delta }}{{G}}_1} \hfill & = \hfill & {\left( {{\mathrm{1}}{\mathrm{/}}{\mathrm{2}}{{E}}_{{{\mathrm{O}}}_2}^{{{\mathrm{DFT}}}} - \left( {{{E}}_{{{n}}_{{\mathrm{s}}}}^{{{\mathrm{DFT}}}} - {{E}}_{{{n}} - 1_{{\mathrm{s}}}}^{{{\mathrm{DFT}}}}} \right)} \right)} \hfill \\ {} \hfill & {} \hfill & { - \left( {{\mathrm{1}}{\mathrm{/}}{\mathrm{2}}{{E}}_{{{\mathrm{O}}}_2}^{{{\mathrm{DFT}}}} - \left( {{{E}}_{{{n}}_{{\mathrm{b}}}}^{{{\mathrm{DFT}}}} - {{E}}_{{{n}} - 1_{{\mathrm{b}}}}^{{{\mathrm{DFT}}}}} \right)} \right)} \hfill \\ {} \hfill & = \hfill & {\left( {{{E}}_{{{n}}_{{\mathrm{b}}}}^{{{\mathrm{DFT}}}} - {{E}}_{{{n}} - 1_{{\mathrm{b}}}}^{{{\mathrm{DFT}}}}} \right) - \left( {{{E}}_{{{n}}_{{\mathrm{s}}}}^{{{\mathrm{DFT}}}} - {{E}}_{{{n}} - 1_{{\mathrm{s}}}}^{{{\mathrm{DFT}}}}} \right)} \hfill \\ {} \hfill & = \hfill & {{\mathrm{\Delta }}{{E}}_{{{\mathrm{O}}},{{\mathrm{bulk}}}}^{{{\mathrm{DFT}}}} - {\mathrm{\Delta }}{{E}}_{{{\mathrm{O}}},{{\mathrm{s}}}}^{{{\mathrm{DFT}}}}} \hfill \end{array}$$

The site-fraction of surface oxygen vacancies (*Γ*_s_) can be written in terms of Δ*G*_2_ as

10 $${\mathrm{\Delta }}{{G}}_2 + 2{{E}}_{{\mathrm{F}}}\left( {{{{\boldsymbol{\Gamma}} }}_{{\mathrm{s}}}} \right) = - {{k}}_{{\mathrm{B}}}{{T}}{\mathrm{ln}}\left( {\frac{{{{\boldsymbol{\Gamma}}}_{{\mathrm{s}}} \cdot \left( {{{P}}_{{{\mathrm{O}}}_2}^{{{\mathrm{gas}}}}} \right)^{1/2}}}{{1 - {{\boldsymbol{\Gamma}} }_{{\mathrm{s}}}}}} \right)$$

Subtracting Eq. (7) from Eq. (10) and rearranging, with the use of Eq. (9) gives:

11 $$\begin{array}{*{20}{l}} {\frac{{{{{\boldsymbol{\Gamma}}}}_{\mathrm{s}}}}{{1 - {{\boldsymbol{\Gamma}} }_{\mathrm{s}}}}} \hfill & = \hfill & {\frac{{x_{\mathrm{v,bulk}}}}{{1 - x_{\mathrm{v,bulk}}}}{\mathrm{exp}}\left[ { - \left( {{\mathrm{\Delta }}G_2 - {\mathrm{\Delta }}G_1} \right){\mathrm{/}}k_{\mathrm{B}}T} \right]} \hfill \\ {} \hfill & = \hfill & {\frac{{x_{\mathrm{v,bulk}}}}{{1 - x_{\mathrm{v,bulk}}}}{\mathrm{exp}}\left[ { - \left( {{\mathrm{\Delta }}E_{\mathrm{O,bulk}}^{\mathrm{DFT}} - {\mathrm{\Delta }}E_{\mathrm{O,s}}^{\mathrm{DFT}}} \right){\mathrm{/}}k_{\mathrm{B}}T} \right]} \hfill \end{array}$$

From DFT calculations, we get that vacancy formation on CoO_2_ surface of LSC-50 is 0.21 to 0.8 eV/vacancy more favorable than the bulk (the range is due to the wide range of vacancy concentrations on the CoO_2_ surface and the strong concentration dependence of the vacancy energetics), while unfavorable by 0.5 eV/vacancy for SrO-terminated LSC-50 (relative to bulk). Based on these value, taking Mizusaki et al.^41^ experiment data for LSC-50 bulk vacancy concentrations, at typical SOFC conditions (650 °C, 0.2 atm) CoO_2_ surface would have 0.28 (per O-site) vacancies, while SrO-termination of LSC-50 would have 5.99 × 10^−5^ vacancies (per Co-site). Since the high oxygen vacancy concentration will have strong vacancy interaction, we write *E*_Form_(V_O_) as a linear function of oxygen vacancy concentration for BO_2_ surface, with a DFT calculated linear dependence on oxygen vacancy concentration of 5.48 eV (per unit concentration) (see Supplementary Table 8). This interaction parameter is estimated by fitting to DFT calculations at surface vacancy concentrations from 0.125 to 0.25 (per O-site), which are close to the final predicted value of 0.28. These calculations therefore should provide a good guide for BO_2_ surface oxygen vacancy interactions. Concentrations of surface species such as *O_2_ and *O can be similarly calculated, Supplementary Table 9 lists the assumptions about degrees of freedom used for calculating concentrations of each surface species.

Consider another example for the reaction of bulk vacancy and surface *O,

12 $$O_{\mathrm{s}}^X \to {\mathrm{V}}_{{\mathrm{O}}_{\mathrm{s}}}^{\cdot\cdot } + 2{\mathrm{e}}^ - + {^{\ast}}\mathrm{O}$$

We use the vibration model in Supplementary Table 9 to calculate the reaction energy. The charge changing for the reaction can be calculated Fig. 2 and Supplementary Table 4.

13 $$\begin{array}{*{20}{l}} {{\mathrm{\Delta }}G_3} \hfill & = \hfill & {\left( {1{\mathrm{/}}2\left( {E_{n + 1_{\mathrm{s}}}^{DFT} - E_{n_{\mathrm{s}}}^{\mathrm{DFT}} + E_{\mathrm{1,DFT}}^{\mathrm{don}}} \right)} \right.} \hfill \\ {} \hfill & {} \hfill & {\left. { - \left( {E_{n_{\mathrm{b}}}^{\mathrm{DFT}} - E_{n - 1_{\mathrm{b}}}^{\mathrm{DFT}} + E_{\mathrm{2,DFT}}^{\mathrm{don}}} \right)} \right)} \hfill \\ {} \hfill & {} \hfill & { + \mathop {\sum }\limits_{i = 1}^{\mathrm{2,s}} k_{\mathrm{B}}T{\mathrm{ln}}\left( {2{\mathrm{sinh}}\left( {\theta _{\mathrm{E},i}{\mathrm{/}}2T} \right)} \right)} \hfill \\ {} \hfill & {} \hfill & { - \mathop {\sum }\limits_{i = 1}^{\mathrm{3,b}} k_{\mathrm{B}}T{\mathrm{ln}}\left( {2{\mathrm{sinh}}\left( {\theta _{\mathrm{E},i}{\mathrm{/}}2T} \right)} \right)} \hfill \\ {} \hfill & {} \hfill & { - {\mathrm{\Delta }}{{h}}_{{\mathrm{O}}}^{{{\mathrm{corr}}}}} \hfill \end{array}$$

$E_{1,\mathrm{DFT}}^{\mathrm{don}}$ and $E_{2,\mathrm{DFT}}^{\mathrm{don}}$ is the donation correction for two DFT calculations. The site-fraction of surface oxygen vacancies (*Γ*_s_) and surface *O $\left( {{{{\boldsymbol{\Gamma}} }}_{{\mathrm{sO}}_2}} \right)$ can be written in terms of Δ*G*_3_ as

14 $$\Delta G_3 + 2E_{\mathrm{F}}\left( {{{\boldsymbol{\Gamma}} }}_{\mathrm{s}} \right) = - k_{\mathrm{B}}T{\mathrm{ln}}\left( {\frac{{{{{\boldsymbol{\Gamma}} }}_{\mathrm{s}} \cdot {{{\boldsymbol{\Gamma}} }}_{{\mathrm{sO}}_2}}}{{\left( {1 - {{{\boldsymbol{\Gamma}} }}_{\mathrm{s}}} \right) \cdot \left( {1 - {{{\boldsymbol{\Gamma}} }}_{{\mathrm{sO}}_2}} \right)}}} \right).$$

### Oxygen adsorption rates from kinetic theory of gases

In the following section, we describe calculations for pre-exponential factors (*k*_*i*_) of chemisorption of oxygen molecules on the surfaces of a cathode, using transition state theory. Oxygen chemisorption involves a significant change in the degrees of freedom from the initial (gaseous) state to the transition state, and assumptions about the nature of transition state have been made in order to quantify the pre-exponential factors. Below we state the assumptions we make about the nature of chemisorbed transition state, and use them to calculate the pre-exponential factors for immobile (C,I) and mobile (C,M) type chemisorption. These give different pre-exponential factors, $k_{\mathrm{ads}}^{\mathrm{I}}$ (immobile) and $k_{\mathrm{ads}}^{\mathrm{M}}$ (mobile) as derived below. The surface-site-specific choices of mobile (C,M) or immobile (C,I) transition state for O_2_ adsorption are also listed in column headers of Supplementary Table 2(mechanisms of SrO termination) and Supplementary Table 5 (mechanisms of CoO_2_ termination).

The general framework for treating chemisorption of gas molecules is taken from the books of Chorkendorff et al.^55^ (chapter 3) and Newman et al.^56^. Consider a cathode surface with *M* sites, total area *A* (thus having *N*_0_=*M*/*A* sites per unit area) in presence of an ideal O_2_ gas at a constant (*T*, $P_{{{\mathrm{O}}_2}}^{\mathrm{gas}}$). Oxygen molecules will adsorb on different surface sites, for example, the surface Co of a CoO_2_-terminated LSC cathode. For a particular type of adsorption site, out of total *M* such sites, let us assume *M*^/^ sites are free for chemisorption (or having available site fraction, Γ_*_ = *M*′/*M*). According to transition state theory, the forward rate of chemisorption can be expressed assuming a chemical equilibrium between the initial state (O_2_ in gas) and the transition state (denoted by a superscript #) for chemisorption. The forward rate (*r*_f_) can be expressed as a product of an attempt frequency at the barrier and concentration of molecules in transition state:

15 $${r}_{\mathrm{f}} = {{\nu }}\frac{{{N}}}{{{M}}}$$

where, ***ν*** is the attempt frequency along the reaction coordinate, *N* is the number of O_2_ in the transition state, *M* is the number of available surface sites for physisorption.

It is useful to express *N* in terms of the partition functions of the initial $\left( {P_{{{\mathrm{O}}_2}}^{\mathrm{gas}}} \right)$ and transition state (*q*^#^), as it can enable calculation while rigorously including all the relevant physics of the adsorption of O_2_ molecules. For a given system of *N* indistinguishable gas molecules (such as pure O_2_) the total partition function of the system is given by $Q = \frac{{q^N}}{{N!}}$, where *q* is the total partition function of individual molecule in transition state, expressed as the product of partition functions of constituent degrees of freedom (typically, *q* = *q*_trans_ × *q*_rot_ × *q*_vib_ × *q*_electronic_ where *q*_trans_, *q*_rot_, *q*_vib_, *q*_electronic_ denote translational, rotational, vibrational and electronic partition functions respectively). The chemical potential of this gas can be expressed in terms of the total individual partition function *q*,

16 $$\begin{array}{*{20}{l}} {{{\mu }}_{{\mathrm{O}}_2}} \hfill & = \hfill & { - {{k}}_{{\mathrm{B}}}{{T}}\left[ {\frac{{\partial {\mathrm{ln}}{{Q}}}}{{\partial {{N}}_{{\mathrm{O}}_2}^{{{\mathrm{gas}}}}}}} \right]_{V,{{T}}}} \hfill \\ {} \hfill & = \hfill & { - {{k}}_{{\mathrm{B}}}{{T}}\left[ {\frac{{\partial {\mathrm{ln}}({{q}}^{{N}}/{{N}}!)}}{{\partial {{N}}_{{\mathrm{O}}_2}^{{{gas}}}}}} \right]_{V,{{T}}}} \hfill \\ {} \hfill & \approx \hfill & { - {{k}}_{{\mathrm{B}}}{{T}}{\mathrm{ln}}\left( {\frac{{{q}}}{{{{N}}_{{\mathrm{O}}_2}^{{{\mathrm{gas}}}}}}} \right)_{V,{{T}}}} \hfill \end{array}$$

where, we have used Stirling’s approximation (ln(*N*!) = *N* · ln(*N*) − *N*).

The partition function for oxygen molecules in chemisorption transition state can be similarly calculated. If *N*^#^ is the number of O_2_-chemisorption species transition state, the total partition function (for *N*^#^ molecules) and respective chemical potential *μ*^#^ is,

17 $$\begin{array}{*{20}{l}} {{{Q}}^{\#} } \hfill & = \hfill & {\frac{{{{M}}^{\prime} !}}{{{{N}}_{{\mathrm{O}}_2}^{\#} !\left( {{{M}}^{\prime} - {{N}}^{\#} } \right)!}}({{q}}^{\#} )^{{{N}}^{\#} } \Rightarrow {{\mu }}_{{{O}}_2}} \hfill \\ {} \hfill & = \hfill & { - {{k}}_{{\mathrm{B}}}{{T}}\left[ {\frac{{\partial {\mathrm{ln}}{{Q}}}}{{\partial {{N}}^{\#} }}} \right]_{V,{{T}}}} \hfill \\ {} \hfill & = \hfill & { - {{k}}_{\mathrm{B}}{{T}}\left[ {\left( {{{M}}^{\prime} - {{N}}^{\#} } \right)\frac{{{{q}}^{\#} }}{{{{N}}^{\#} }}} \right]} \hfill \end{array}$$

where, we have used Eq. (15). Thus for an equilibrium between oxygen gas and oxygen molecules in transition state, we can write $\mu _{{{\mathrm{O}}_2}}^{\mathrm{gas}} = \mu _{{{\mathrm{O}}_2}}^\#$, and it can be shown that,

18 $$\begin{array}{*{20}{l}} {{{\mu }}_{{\mathrm{{O}}_2}}^{{\mathrm{gas}}}} \hfill & = \hfill & {{{\mu }}_{{\mathrm{{O}}_2}}^\# \Rightarrow \frac{{{{N}}^\# }}{{{{N}}_{{\mathrm{{O}}_2}}^{{\mathrm{gas}}}}}} \hfill \\ {} \hfill & = \hfill & {\left( {{{M}}^{\prime} - {{N}}^\# } \right)\frac{{{{q}}^\# }}{{{{q}}_{{\mathrm{O}}_2}^{{\mathrm{gas}}}}}} \hfill \\ {} \hfill & = \hfill & {{{M}}\left( {{{{\boldsymbol{\Gamma}} }}_ \ast - {{\theta }}^\# } \right)\frac{{{{q}}^\# }}{{{{q}}_{{\mathrm{{O}}_2}}^{{\mathrm{gas}}}}}} \hfill \\ {} \hfill & \approx \hfill & {{{M}}{{{\boldsymbol{\Gamma}} }}_ \ast \frac{{{{q}}^\# }}{{{{q}}_{{\mathrm{{O}}_2}}^{{\mathrm{gas}}}}}} \hfill \end{array}$$

Where, *θ*^#^ is the coverage of O_2_ species in transition state, which are likely to be small, compared to the available adsorption sites (Γ_*_ = *M*′/*M*) and can be neglected. Substituting Eq. (18) into Eq. (15) yields (assuming O_2_ as an ideal gas and using $P_{{\mathrm{O}}_2}^{\mathrm{gas}}V = N_{{\mathrm{O}}_2}^{\mathrm{gas}}k_{\mathrm{B}}T$,)

19 $${{r}}_{\mathrm{f}} = {{\nu }}\frac{{{{N}}^{\#} }}{{{M}}} = {{v}}\frac{{{{N}}_{{\mathrm{O}}_2}^{{\mathrm{gas}}}}}{{{M}}}{{M}}{{{\boldsymbol{\Gamma}} }}_ \ast \frac{{{{q}}^{\#} }}{{{{q}}_{{\mathrm{O}}_2}^{\mathrm{gas}}}} = {{v}}{{{\boldsymbol{\Gamma}} }}_ \ast \frac{{{{P}}_{{\mathrm{O}}_2}^{\mathrm{gas}}V}}{{{{k}}_{{\mathrm{B}}}{{T}}}}\frac{{{{q}}^{\#} }}{{{{q}}_{{\mathrm{O}}_2}^{\mathrm{gas}}}}$$

Calculation of the partition function of oxygen gas is straightforward (expressed as $q_{{\mathrm{O}}_2}^{\mathrm{gas}} = q_{\mathrm{trans}}^{3\mathrm{D}}q_{\mathrm{rot}}^{\mathrm{gas}}q_{\mathrm{vib}}^{\mathrm{gas}}q_{\mathrm{elec}}^{\mathrm{gas}}$), where, $q_{\mathrm{trans}}^{\mathrm{3D}}$, $q_{\mathrm{rot}}^{\mathrm{gas}}$, $q_{\mathrm{vib}}^{\mathrm{gas}}$, $q_{\mathrm{elec}}^{\mathrm{gas}}$, denote 3D translational, rotational and vibrational and electronic partition functions of oxygen gas.

### Immobile transition state for chemisorption

For the chemisorption transition state of the immobile type, we assume the following about its nature, which allows us to estimate the partition function of transition state.

1. We assume that the transition state of O_2_ chemisorption is immobile and only retains the vibrational degree of freedom from the 3D-gas phase
2. We assume the transition state has gained the lattice-vibrational degrees of freedom of the final chemisorbed state, except the ‘reaction coordinate’ degree of freedom. Thus we approximate the partition function of transition state *q*^#^ assuming 4 modes of vibration of lattice oxygen, 1 mode of O–O vibration retained from gas, and the reaction coordinate.

Under these assumptions, *q*^#^ becomes:

20 $${{q}}^\# = {{q}}_{\text{reaction-coord}}{{q}}_{{\mathrm{vib}}}^{{\mathrm{gas}}}\left( {{{q}}_{{\mathrm{vib}}}^{{\mathrm{lattice}}}} \right)^4{{q}}_{{\mathrm{elec}}}^\# = \frac{{{{k}}_{\mathrm{B}}{{T}}}}{{{{h\nu }}}}{{q}}_{{\mathrm{vib}}}^{{\mathrm{gas}}}\left( {{{q}}_{{\mathrm{vib}}}^{{\mathrm{lattice}}}} \right)^4{{q}}_{{\mathrm{elec}}}^\#$$

Thus, forward rate of oxygen chemisorption, from combining Eqs. (19) and (20) is,

21 $$\begin{array}{*{20}{l}} {{{r}}_{\mathrm{f}}} \hfill & = \hfill & {{{v}}{{{\boldsymbol{\Gamma}} }}_ \ast \frac{{{{P}}_{\mathrm{{O}}_2}^{{\mathrm{gas}}}V}}{{{{k}}_{\mathrm{B}}{{T}}}}\frac{{{{q}}^\# }}{{{{q}}_{{\mathrm{O}}_2}^{{\mathrm{gas}}}}}} \hfill \\ {} \hfill & = \hfill & {{{{\boldsymbol{\Gamma}}}}_ \ast \times \frac{{{{P}}_{{\mathrm{O}}_2}^{{\mathrm{gas}}}V}}{{{{k}}_{\mathrm{B}}{{T}}}} \times \frac{{{{k}}_{\mathrm{B}}{{T}}}}{{{h}}} \times \frac{{{{q}}_{{\mathrm{vib}}}^{{\mathrm{gas}}}\left( {{{q}}_{{\mathrm{vib}}}^{{\mathrm{lattice}}}} \right)^4{{q}}_{{\mathrm{elec}}}^\# }}{{{{q}}_{{\mathrm{trans}}}^{3{\mathrm{D}}}{{q}}_{{\mathrm{rot}}}^{{\mathrm{gas}}}{{q}}_{{\mathrm{vib}}}^{{\mathrm{gas}}}{{q}}_{{\mathrm{elec}}}^{{\mathrm{gas}}}}}} \hfill \end{array}$$

The ratio of electronic partition functions of the transition and initial state $\left( {q_{\mathrm{elec}}^\# {\mathrm{/}}q_{\mathrm{elec}}^{\mathrm{gas}}} \right)$ can be approximated to be ${\mathrm{e}}^{ - {\mathrm{\Delta }}G_{\mathrm{eff}}/k_{\mathrm{B}}T}$where, ${\mathrm{\Delta }}G_{\mathrm{eff}} = ({\mathrm{\Delta }}G_{\mathrm{f}}^0 - (1 - \beta ){\mathrm{\Delta }}E)$ as described above, is the difference between the electronic energy of the transition state and the initial (gas) state, estimated using DFT. For an ideal gas, the forward rate then becomes,

22 $$\begin{array}{*{20}{l}} {{{r}}_{\mathrm{f}}} \hfill & = \hfill & {\frac{{{{M}}{{{\boldsymbol{\Gamma}} }}_ \ast }}{{{M}}}\frac{{{{P}}_{{\mathrm{O}}_2}^{{\mathrm{gas}}}V}}{{{{k}}_{\mathrm{B}}{{T}}}} \times \frac{{{{k}}_{\mathrm{B}}{{T}}}}{{{h}}}} \hfill \\ {} \hfill & {} \hfill & { \times {\textstyle{1 \over {V\left( {2{{\pi mk}}_{\mathrm{B}}{{T}}} \right)^{\frac{3}{2}}{\mathrm{/}}{{h}}^3}}}\left( {{\textstyle{{{{A}}\left( {2{{\pi mk}}_{\mathrm{B}}{{T}}} \right){\mathrm{/}}{{h}}^2} \over {{{A}}\left( {2{{\pi mk}}_{\mathrm{B}}{{T}}} \right){\mathrm{/}}{{h}}^2}}}} \right){\textstyle{{{{q}}_{{\mathrm{vib}}}^{{\mathrm{gas}}}\left( {{{q}}_{{\mathrm{vib}}}^{{\mathrm{lattice}}}} \right)^4} \over {{{q}}_{{\mathrm{rot}}}^{{\mathrm{gas}}}{{q}}_{{\mathrm{vib}}}^{{\mathrm{gas}}}}}}{\mathrm{e}}^{ - \frac{{{\mathrm{\Delta }}{{G}}_{{\mathrm{eff}}}}}{{{{k}}_{\mathrm{B}}{{T}}}}}} \hfill \\ {} \hfill & = \hfill & {\frac{1}{{{{N}}_0\sqrt {2{{\pi mk}}_{\mathrm{B}}{{T}}} }} \times \frac{{\left( {{{q}}_{{\mathrm{vib}}}^{{\mathrm{lattice}}}} \right)^4}}{{{{q}}_{{\mathrm{trans}}}^{2{\mathrm{D}}}{{q}}_{{\mathrm{rot}}}^{{\mathrm{gas}}}}} \times {{P}}_{{\mathrm{O}}_2}^{{\mathrm{gas}}} \times {{{\boldsymbol{\Gamma}} }}_ \ast \times {\mathrm{e}}^{ - \frac{{{\mathrm{\Delta }}{{G}}_{{\mathrm{eff}}}}}{{{{k}}_{\mathrm{B}}{{T}}}}}} \hfill \end{array}$$

where, *N*_0_, *A* are as defined above, and $q_{\mathrm{trans}}^{2\mathrm{D}} = A(2\pi mk_{\mathrm{B}}T){\mathrm{/}}h^2$ is the partition function for 2D ideal gas, normalized per adsorption site (hence divided by *M*).

The forward rate of chemisorption reaction $\left( {\mathrm{O}_2 + \ast \to \mathrm{O}_2^{\mathrm{ads}}} \right)$ via immobile transition state from the thermo-kinetic model is given by,

23 $${{r}}_{\mathrm{f}} = {{k}}_{{\mathrm{ads}}}^{\mathrm{I}} \times {{P}}_{{\mathrm{O}}_2}^{{\mathrm{gas}}} \times {{{\boldsymbol{\Gamma}} }}_ \ast \times {\mathrm{e}}^{ - \frac{{{\mathrm{\Delta }}{{G}}_{{\mathrm{eff}}}}}{{{{k}}_{\mathrm{B}}{{T}}}}}$$

where, $k_{\mathrm{ads}}^{\mathrm{I}}$ is the pre-exponential factor for oxygen chemisorption via immobile transition state. Clearly,

24 $$k_{\mathrm{ads}}^{\mathrm{I}} = \frac{1}{{N_0\sqrt {2\pi mk_{\mathrm{B}}T} }} \times \frac{{\left( {q_{\mathrm{vib}}^{\mathrm{lattice}}} \right)^4}}{{q_{\mathrm{trans}}^{2\mathrm{D}}q_{\mathrm{rot}}^{\mathrm{gas}}}}$$

As discussed in “Methods” section (main text), an Einstein temperature of *T*_θ_ = 275 K, gives accurate expression for enthalpy and entropy of lattice oxygen in equilibrium with the gas. Using this Einstein temperature, the vibrational partition function for lattice-O at *T* = 650 °C is approximately 3.34 (unitless). At *T* = 650 °C, the value of pre-exponential factor for oxygen chemisorption via immobile transition state, $k_{\mathrm{ads}}^{\mathrm{I}}$ = 0.65 m s kg^−1^. Values of various terms of above equation are in Supplementary Table 8.

### Mobile transition state for chemisorption

For the chemisorption transition state of the mobile type, we assume the following about its nature, which allows us to estimate the partition function of transition state.

1. We assume that the transition state of O_2_ chemisorption is relatively mobile, and has retained both the rotational and vibrational degrees of freedom from the 3D-gas phase, but lost all 3-translational modes
2. We assume the transition state has gained 2 lattice-vibrational degrees of freedom of the final chemisorbed state, and a ‘reaction coordinate’ degree of freedom. Thus we approximate the partition function of transition state *q*^#^ assuming 2 modes of vibration of lattice oxygen, 1 mode of O–O vibration and 2 rotational modes retained from gas, and the reaction coordinate.

Under these assumptions, similar to Eq. (22), *r*_f_ becomes:

25 $$\begin{array}{*{20}{l}} {{{r}}_{\mathrm{f}}} \hfill & = \hfill & {\frac{{{{M}}{{{\boldsymbol{\Gamma}} }}_ \ast }}{{{M}}}\frac{{{{P}}_{{\mathrm{O}}_2}^{{\mathrm{gas}}}V}}{{{{k}}_{\mathrm{B}}{{T}}}} \times \frac{{{{k}}_{\mathrm{B}}{{T}}}}{{{h}}}} \hfill \\ {} \hfill & {} \hfill & { \times {\textstyle{1 \over {V\left( {2{{\pi mk}}_{\mathrm{B}}{{T}}} \right)^{\frac{3}{2}}/{{h}}^3}}}\left( {{\textstyle{{{{A}}\left( {2{{\pi mk}}_{\mathrm{B}}{{T}}} \right){\mathrm{/}}{{h}}^2} \over {{{A}}\left( {2{{\pi mk}}_{\mathrm{B}}{{T}}} \right){\mathrm{/}}{{h}}^2}}}} \right){\textstyle{{{{q}}_{{\mathrm{rot}}}^{{\mathrm{gas}}}{{q}}_{{\mathrm{vib}}}^{{\mathrm{gas}}}\left( {{{q}}_{{\mathrm{vib}}}^{{\mathrm{lattice}}}} \right)^4} \over {{{q}}_{{\mathrm{rot}}}^{{\mathrm{gas}}}{{q}}_{{\mathrm{vib}}}^{{\mathrm{gas}}}}}}{\mathrm{e}}^{ - \frac{{{\mathrm{\Delta }}{{G}}_{{\mathrm{eff}}}}}{{{{k}}_{\mathrm{B}}{{T}}}}}} \hfill \\ {} \hfill & = \hfill & {\frac{1}{{{{N}}_0\sqrt {2{{\pi mk}}_{\mathrm{B}}{{T}}} }} \times \frac{{\left( {{{q}}_{{\mathrm{vib}}}^{{\mathrm{lattice}}}} \right)^4}}{{{{q}}_{{\mathrm{trans}}}^{2{\mathrm{D}}}}} \times {{P}}_{{\mathrm{O}}_2}^{{\mathrm{gas}}} \times {{{\boldsymbol{\Gamma}} }}_ \ast \times {\mathrm{e}}^{ - \frac{{{\mathrm{\Delta }}{{G}}_{{\mathrm{eff}}}}}{{{{k}}_{\mathrm{B}}{{T}}}}}} \hfill \end{array}$$

The forward rate of chemisorption reaction $\left( {\mathrm{O}_2 + \ast \to \mathrm{O}_2^{\mathrm{ads}}} \right)$ via immobile transition state from the thermo-kinetic model is given by,

26 $${{r}}_{\mathrm{f}} = {{k}}_{{\mathrm{ads}}}^{\mathrm{M}} \times {{P}}_{{\mathrm{O}}_2}^{{\mathrm{gas}}} \times {{{\boldsymbol{\Gamma}}}}_ \ast \times {\mathrm{e}}^{ - \frac{{{\mathrm{\Delta }}{{G}}_{{\mathrm{eff}}}}}{{{{k}}_{\mathrm{B}}{{T}}}}}$$

where, $k_{\mathrm{ads}}^{\mathrm{M}}$ is the pre-exponential factor for oxygen chemisorption via a mobile transition state. Thus we can write,

27 $${{k}}_{{\mathrm{ads}}}^{\mathrm{M}} = \frac{1}{{{{N}}_0\sqrt {2{{\pi mk}}_{\mathrm{B}}{{T}}} }} \times \frac{{\left( {{{q}}_{{\mathrm{vib}}}^{{\mathrm{lattice}}}} \right)^4}}{{{{q}}_{{\mathrm{trans}}}^{2{\mathrm{D}}}}}$$

At *T* = 650 °C, the value of pre-exponential factor for oxygen chemisorption via mobile transition state, $k_{\mathrm{ads}}^{\mathrm{M}}$ ~ 17.55 m·s·kg^−1^. Values of various terms of the above equation are in Supplementary Table 8.

### Adsorption limit on *R*_0_, *K*_tr_ and oxygen exchange

Here we calculate the fundamental upper limits on *K*_tr_ (m·s^−1^) as well as ORR rate (moles of O_2_·m^2^·s^−1^) for any perovskite oxide cathode material. Recent work by De Souza et al.^57^ shows the k* limited by arrival from the gas phase using a simplified kinetic model that requires proposing a critical incident kinetic energy needed to incorporate an arriving O_2_, *E*_ct_. Their work predicts a k* maximum about 10^0^ cm·s^−1^, which occurs when *E*_ct_ = 0. Our more detailed kinetic model does not require positing a value of *E*_ct_ and matches the approximate k* upper limit in De Souza et al.^57^ when their critical energy is *E*_ct_ = 0.3 eV, which seems a physically reasonable value and lends support to both approaches. To represent typical IT-SOFC working conditions, we calculate the limiting ORR rates and upper limits on *K*_tr_ for a temperature range of 500–750 °C, at a 0.2 atm oxygen partial pressure. In these conditions, typical SOFC cell voltages are in the range of 1–1.5 V. Let us assume that the entire driving force for the fuel cell is represented in the deviation from equilibrium of the ORR reaction, $\frac{1}{2}{\mathrm{O}}_2 + {\mathrm{V}}_{\mathrm{O}}^{\cdot\cdot} + 2{\mathrm{e}}^ - \to {\mathrm{O}}_{{\mathrm{O}}_{\mathrm{b}}}^X$ where ${\mathrm{V}}_{\mathrm{O}}^{..}$ is oxygen vacancy and ${\mathrm{O}}_{{\mathrm{O}}_{\mathrm{b}}}^X$ is lattice oxygen in the cathode.

From Eqs. (5) and (7), we have non-configurational Gibbs free energy Δ*G* = −Δ*G*_1._ Δ*G*_1_ represents an ideal, non-configurational Gibb’s free energy change of the formation of oxygen vacancy.

Therefore, at the equilibrium state, we can write the cathode reaction total Gibbs free energy $\Delta G_1^ {\ast}$ and cathode potential *E*_1_ as,

28 $$\begin{array}{*{20}{l}} {\Delta G_1^ {\ast} } \hfill & = \hfill & { - 2FE_1} \hfill \\ {} \hfill & = \hfill & { - \Delta G_1 - 2E_{\mathrm{F}}\left( {x_{\mathrm{v,bulk,eq}}} \right)} \hfill \\ {} \hfill & {} \hfill & { + k_{\mathrm{B}}T{\mathrm{ln}}\left( {\frac{{1 - x_{\mathrm{v,bulk,eq}}}}{{\left( {P_{{\mathrm{O}}_2}^{\mathrm{gas}}} \right)^{\frac{1}{2}}x_{\mathrm{v,bulk,eq}}}}} \right)} \hfill {} \hfill & = \hfill & 0 \hfill \end{array}$$

*F* is Faraday constant. Similarly, at the non-equilibrium state, we have the fixed oxygen pressure and new bulk vacancy concentration *x*_v,bulk_, the cathode reaction total Gibbs free energy $\Delta G_2^ \ast$ and cathode potential *E*_2_ is calculated as,

29 $$\begin{array}{*{20}{l}} {{\mathrm{\Delta }}{{G}}_2^ \ast } \hfill & = \hfill & { - 2{{FE}}_2} \hfill \\ {} \hfill & = \hfill & { - {\mathrm{\Delta }}{{G}}_2 - 2{{E}}_{\mathrm{F}}\left( {{{x}}_{{\mathrm{v,bulk}}}} \right)} \hfill \\ {} \hfill & {} \hfill & { + {{k}}_{\mathrm{B}}{{T}}{\mathrm{ln}}\left( {\frac{{1 - {{x}}_{{\mathrm{v,bulk}}}}}{{\left( {{{P}}_{{\mathrm{O}}_2}^{{\mathrm{gas}}}} \right)^{\frac{1}{2}}{{x}}_{{\mathrm{v,bulk}}}}}} \right)} \hfill {} \hfill & = \hfill & 0 \hfill \end{array}$$

At the small overpotential *η*(V),

30 $$\begin{array}{*{20}{l}} {{\eta }} \hfill & = \hfill & {{{E}} - {{E}}_{{\mathrm{eq}}}} \hfill \\ {} \hfill & = \hfill & {{{E}}_2 - {{E}}_1} \hfill \\ {} \hfill & = \hfill & { - \frac{{{{E}}_{\mathrm{F}}\left( {{{x}}_{{\mathrm{v,bulk}}}} \right)}}{{{F}}} + \frac{{{{k}}_{\mathrm{B}}{{T}}}}{{2{{F}}}}{\mathrm{ln}}\left( {\frac{{1 - {{x}}_{{\mathrm{v,bulk}}}}}{{\left( {{{P}}_{{\mathrm{O}}_2}^{{\mathrm{gas}}}} \right)^{\frac{1}{2}}{{x}}_{{\mathrm{v,bulk}}}}}} \right)} \hfill \\ {} \hfill & {} \hfill & { + \frac{{{{E}}_{\mathrm{F}}\left( {{{x}}_{{\mathrm{v,bulk,eq}}}} \right)}}{{{F}}} - \frac{{{{k}}_{\mathrm{B}}{{T}}}}{{2{{F}}}}{\mathrm{ln}}\left( {\frac{{1 - {{x}}_{{\mathrm{v,bulk,eq}}}}}{{\left( {{{P}}_{{\mathrm{O}}_2}^{{\mathrm{gas}}}} \right)^{\frac{1}{2}}{{x}}_{{\mathrm{v,bulk,eq}}}}}} \right)} \hfill \end{array}$$

$\left[ {{\mathrm{O}}_{{\mathrm{O}}_{\mathrm{b}}}^X} \right]$ is the concentration of lattice-O ions

31 $$\left[ {\mathrm{O}}_{{\mathrm{O}}_{\mathrm{b}}}^X \right] = 1 - x_{\mathrm{v,bulk}}$$

For LSC-50 cathode, with typical SOFC cathode overpotential of *η* = −0.2 V, *p*O_2_ = 0.2 atm, the term $\left( {\left[ {{\mathrm{O}}_{{\mathrm{O}}_{\mathrm{b}}}^X} \right] - \left[ {{\mathrm{O}}_{{\mathrm{O}}_{\mathrm{b}}}^{X,\mathrm{eq}}} \right]} \right)$ is calculated at *T* = 500 °C, 650 °C, 750 °C, 800 °C, respectively, to be 1168 moles-O/m^3^ (0.015 site-fraction), 2231 moles-O·m^−3^ (0.029 site-fraction), 2929 moles-O·m^−3^ (0.038 site-fraction) and 3299 moles-O·m^−3^ (0.042 site-fraction). Values of *x*_v_ are fitted using the parameters from Mizusaki et al.^41^ for LSC-50. Figure 7 below plots the term ($\left[ {{\mathrm{O}}_{{\mathrm{O}}_{\mathrm{b}}}^X} \right] - \left[ {{\mathrm{O}}_{{\mathrm{O}}_{\mathrm{b}}}^{X,\mathrm{eq}}} \right]$, % O-site fraction) on the x-axis and ORR rates (moles-O_2_·m^−2^·s^−1^) on the *y*-axis. Limiting rates are shown as horizontal lines of the type *y* = *R*_0_ (moles-O_2_·m^−2^·s^−1^), with *R*_0_ of the O_2_ chemisorption step that sets the fundamental upper limit to ORR. Limiting *K*_tr_ is represented by the slope (*m*) of the line of the type *y *= m*x*; in other words, a line which gives the limiting rate at the typical values of driving force $\left( {\left[ {{\mathrm{O}}_{{\mathrm{O}}_{\mathrm{b}}}^X} \right] - \left[ {{\mathrm{O}}_{{{\mathrm{O}}_{\mathrm{b}}}}^{X,\mathrm{eq}}} \right]} \right)$. Plots at three different temperatures are made for the purpose of illustration.

### Overpotential calculations

Based on our surface reaction model we can calculate the cathode overpotential vs. current density, providing another method of comparing our model to experiments. The calculation involves a few steps, which we describe below. First, we obtain *R*_0_ for the material under polarization as follows. From Eq. (30), we can calculate the bulk oxygen vacancy concentration *x*_v_ under polarization. We can then use the bulk oxygen vacancy concentration under polarization to calculate the concentration of surface oxygen adatom *Γ*_Oads_ and surface oxygen vacancy *Γ*_s_. The equilibrium exchange rate from our rate limiting step can then be updated to include polarization effect from the equation *R*_0_(diffusion) = (8 × *D*_V_ × 2*Γ*_s_ × *Γ*_Oads_)/*d*^2^. Now we relate *R*_0_ to the current density and overpotential through the total reaction rate.

The total reaction rate r can be written in terms of equilibrium exchange rate *R*_0_ in Eq. (3a)

32 $${{{\boldsymbol{\Lambda}} }}_{\mathrm{i}} = {{{\boldsymbol{\Lambda}} /}}\lambda_{\mathrm{i}} = k_{\mathrm{B}}T\,{\mathrm{ln}}\left( {x_{\mathrm{v,bulk,eq}}{\mathrm{/}}x_{\mathrm{v,bulk}}} \right)$$

where *λ*_i_ is the stoichiometric ratio between reaction i and overall reaction under quasi-equilibrium conditions, detail see Adler et al.^11^. *Λ* is the driving force for overall reaction.

We can write the current density as *j* = 4*e* · *r*/*S*. The unit of *r* is O_2_·Co^−1^·s^−1^. *S* is the area of one Co site. *e* is the charge of one electron.

Based on the Eqs. (3a), (30) and (32), we can calculate the activation potential of the overpotential curve. The total overpotential consists of activation polarization, ohmic polarization and concentration polarization.^58,59^ For a dense thin film, the concentration polarization is assumed to be negligible, and for the comparisons in this work we use experimental data from which the ohmic polarization has been subtracted.

**Fig. 9:**
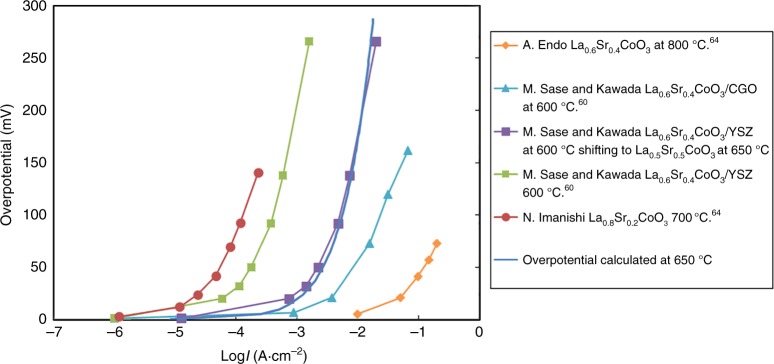
Overpotential versus log of the current density plot. *X*-axis is log of current density *I* (A·cm^−2^). Blue line is our calculated overpotential at 650 °C. Magenta circles line refers to experiment data from N. Imanishi et al.^62^. Black squares and azure triangles refer to M. Sase and Kawada et al.^60^ for La_0.6_Sr_0.4_CoO_3_/yttria-stabilized zirconia (YSZ) and La_0.6_Sr_0.4_CoO_3_/gadolinium doped ceria (CGO). Orange diamonds refers to Akira Endo et al.^63^. To compare our data with experiment data, we shift the experiment data from M. Sase and Kawada for La_0.6_Sr_0.4_CoO_3_/Yttria-stabilized zirconia (YSZ) (black squares) by +1.1 log units in current density to obtain the La_0.6_Sr_0.4_CoO_3_/YSZ “with shifting” (purple squares)^60^ (see text for details on the shifting)

**Fig. 10:**
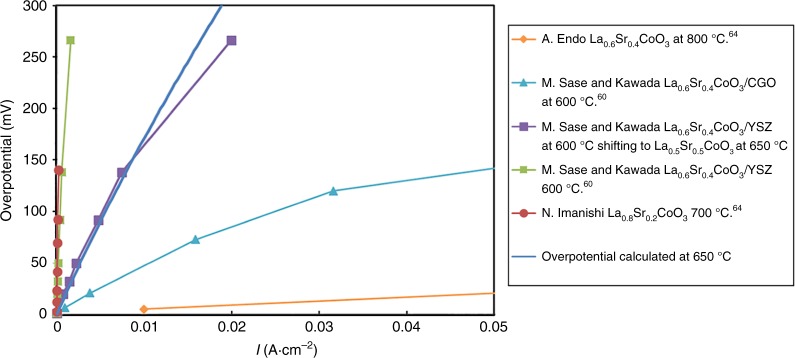
Overpotential versus current density plot. *X*-axis is current density *I* (A·cm^−2^). Blue line is our calculated overpotential at 650 °C. Magenta circles refers to experiment data from N. Imanishi et al.^62^. Black squares and azure triangles refer to M. Sase and Kawada et al.^60^. Orange diamonds refers to Akira Endo et al.^63^. To compare our data with experiment data, we shift the experiment data from M. Sase and Kawada for La_0.6_Sr_0.4_CoO_3_/YSZ (black squares) by +1.1 log units in current density to obtain the La_0.6_Sr_0.4_Co/YSZ “with shifting” (purple squares)^60^ (see text for details on the shifting)

**Fig. 11:**
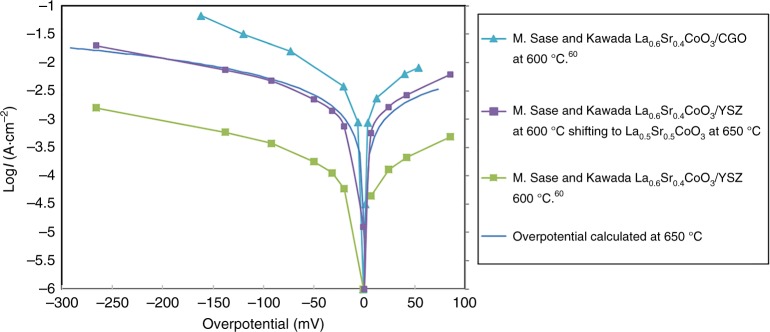
Positive and negative overpotential plot. Blue line is our calculated overpotential at 650 °C. Black squares and azure triangles refer to M. Sase and Kawada et al.^60^. To compare our data with experiment data, we shift the experiment data from M. Sase and Kawada for La_0.6_Sr_0.4_CoO_3_/YSZ (black squares) by +1.1 log units in current density to obtain the La_0.6_Sr_0.4_CoO_3_/YSZ “with shifting” (purple)^60^ (see text for details on the shifting)

The predicted activation polarization values are given in Fig. 9 (overpotential vs. log(I) for cathodic polarization), Fig. 10 (overpotential vs. I for cathodic polarization) and Fig. 11 (log(I) vs. overpotential for both anodic and cathodic polarization). In these figures we include a number of experimental data sets on similar materials under similar conditions. However, we were not able to find any studies under the exact conditions of our modeling. Due to the sensitivity of overpotential to exact composition and temperature, a direct quantitative comparison between our model and these studies is difficult. As our best attempt at such a comparison, we shift the La_0.6_Sr_0.4_Co/YSZ data from Sase and Kawada^60^ (black) by +1.1 log units in current density to obtain the La_0.6_Sr_0.4_CoO_3_/YSZ “with shifting” curves (purple data). This shift is based on the k*temperature dependence and k* difference between La_0.6_Sr_0.4_CoO_3_ and La_0.5_Sr_0.5_CoO_3_ from Egger et al.^7^ Specifically, this data suggests we should expect a shift of +1.1 log units in going from La_0.6_Sr_0.4_CoO_3_ at 600 °C to La_0.5_Sr_0.5_CoO_3_ at 650 °C (which are our modeling conditions), which should lead to an equivalent shift in current density since these measurements on are surface exchange limited films. In comparing our result to that of the shifted curve from Sase and Kawada we see the similar exponential relationship of log(*I*) and overpotential in Fig. 9 and the same decreasing slope in Fig. 10. Given the uncertainties in the experiments and modeling, and the difference of condition, the agreement is very satisfactory. The slope of our prediction in Fig. 9 is slightly smaller than the experiment at high current density. According to the Eq. (30), the overpotential consists of one linear and one log function of a term that changes approximately proportional to oxygen vacancy concentration. At high oxygen vacancy concentration, the linear term will dominate the overpotential. The faster increase of the model as compared to experimental overpotential suggests that the linear term is changing too fast in our model, i.e., too much overpotential is required to create enough vacancies to enable the observed current density. This could be because it is too hard to create oxygen vacancies in our model, particularly at higher oxygen vacancy content. Our model follows that of Mizusaki, et al.^41^ in having oxygen vacancy formation energies increase with increasing oxygen vacancy concentration through filling of electronic states at the Fermi level, which creates a significant coupling between oxygen vacancy formation and the density of states at the Fermi level. Our density of states is from the fitting from Mizusaki et al.^41^, and represented by the bulk vacancy interaction parameter “*a*” in Table I of Mizusaki et al.^41^. However, our vacancy concentration at high current density is beyond any values explored in the experimental data that was fit by Mizusaki et al.^41^. It is therefore possible the bulk vacancy interaction parameter will decrease at high vacancy concentration. That would lead to the decrease of overpotential at high current density, which would match the experiment data in Fig. 9. Further study of the behavior of the vacancy energetics at high vacancy concentration therefore might be of use, but it beyond the scope of this work.

## Supplementary information

Supplementary Information

## Data Availability

The authors declare that the main data supporting the findings of this study are available within the article and its Supplementary Information files. Extra data that support the findings of this study are available from the authors on reasonable request.
