# Supplementary material for: Factors controlling surface oxygen exchange in oxides
Source: Nat Commun. 2019 Mar 22;10:1346. doi: 10.1038/s41467-019-08674-4 (PMC6430818; doi:10.1038/s41467-019-08674-4)
Supplement: Supplementary file 1 — Supplementary Information [file 41467_2019_8674_MOESM1_ESM.pdf]

Supplemental Information

Factors controlling surface oxygen exchange in oxides

Cao et al.

1 Supplementary Note 1

1.1 SrO termination

Neutral building units of surface species of the (001)-SrO termination and their chemical potentials are defined in Supplementary Table 1, and molecular configurations in Figure 2 in the main text. In Supplementary Table 1, the 2nd column defines the chemical potentials of corresponding neutral building units. Here, μ_i^0 is the reference partial molar free energy of the unit i, Γ_i is the fraction of active surface sites (normalized per surface oxygen lattice site) occupied by species i, and E_F is the Fermi Energy relative to its value at full oxygen stoichiometry. Because the materials we are considering ($La_{1-x}Sr_xCoO_{3-\delta}$) are itinerant electronic conductors[1,2], we assume that the electrons can be treated as delocalized charges at the Fermi Level, and that the Fermi Level at the surface is the same as that in the bulk. In Supplementary Table 1, Sr_{br} denotes a bridge site between two nearest-neighbor surface Sr-ions, Sr_{top} and O_{top} denote sites on top of Sr and oxygen respectively. Site: 'Sr_{br}-O_{top}' adsorbs an O_2 molecule to form an ozone-like structure with O-O bond lengths of 1.5Å, 1.44Å and O-O-O bond angle of 109.93 degrees. This configuration is shown in Figure 2 in the main text denoted as '$*O_2(Sr_{br}$-$O_{top})$', as one of the adsorbed oxygen sits on 'Sr_{br}' site and the other occupies O_{top} site.

Supplementary Table 2 shows a 14×14 matrix of various possible mechanisms of oxygen exchange on SrO termination, based on the 14 possible sites of adsorption (rows) and 14 possible sites of dissociation (columns) on a CoO_2 terminated LSC surface. In Supplementary Table 2, the column headers read (*C*,*I*), or (*C*,*M*), and respectively describe our approximations of *mobile* (*M*) and *immobile* (*I*) nature of the transition state

for oxygen chemisorption(C) step; which enable us (as we describe in “Methods “section in the main text) to estimate pre-exponential factors for chemisorption. A dashed line (`-’) in Supplementary Table 2 implies that such a reaction mechanism is unlikely because of site constraints or unphysical molecular processes. For example, only meaningful pathway for the site type [Sr_{top}-Sr_{top}] (*i.e.* a pair of nearest neighbor surface Sr_{top} sites) is simultaneous adsorption and dissociation on [Sr_{top}-Sr_{top}], (denoted A36 in Supplementary Table 2) whereas, all other dissociation options in that column are unphysical, as the molecule has already dissociated while adsorbing on this site. With such a classification, for SrO termination, a total of 41 reaction mechanisms emerge for SrO termination. Amongst these 41 mechanisms, Mechanisms such as A9, A20, A23, A26 involve a barrierless or a low barrier (0.1eV) chemisorption of O_2 on a surface site (such as Sr_{br}, Sr_{top}), followed by diffusion towards a surface vacancy and fast incorporation, and hence are likely to be significant. Other mechanisms have either: i) a slow initial step (example mechanisms: A1, A13, A32-A41) where O_2 (gas) directly adsorbs and simultaneously splits into two 2*O(site), or, ii) have metastable intermediates such as *O(Sr_{br}), *O(Sr_{top}) (example, mechanisms A12-A14), or, iii) With a typical O-O dissociation barrier of 1eV, due to a metastable intermediate, or iv), due to involvement of rare vacancies on SrO surface these mechanisms are likely to be at least 5-6 orders of magnitude slower than key, fast mechanisms such as A9. Key mechanisms on the SrO termination, with an example of a slow mechanism (A39) are listed in Supplementary Table 3 and their energy landscape is depicted in Figure 4 in the main text.

Supplementary Table 1: Definitions of neutral building units of intermediates of the (001) SrO-termination used in this study, and their chemical potentials. Itinerant electronic structure of the LSC material is assumed[1-4], wherein electrons are donated to the Fermi level.

Neutral Building Unit	Chemical potential
$s_{AO} = \left\{ V_{O_S}^{q_S^{\bullet}} + \left(q_S - q_O\right)e^{-} - O_{O_S}^{q_O^{\bullet}} \right\}$ (Isolated vacancy on SrO surface)	$\mu_{S-AO} = \mu^0_{S-AO} + k_B T \ln\left(\Gamma_S \big/ \left(1 - \Gamma_S\right)\right) + \left(q_S - q_O\right) E_F$
$O_2\left(Sr_{br}\right) = \left\{ \left(O_2\right)^{q_{O_2}^{\bullet}}_{Sr-br} + q_{O_2} e^{-} \right\}$ (O_2-adsorbed on Sr-Sr bridge site)	$\mu_{O_2^{Sr-br}} = \mu^0_{O_2^{Sr-br}} + k_B T \ln\left(\Gamma_{O_2^{Sr-br}} \Big/ \left(1 - \Gamma_{O_2^{Sr-br}}\right)\right) + q_{O_2} E_F$
$O_2\left(Sr_{top}\right) = \left\{ \left(O_2\right)^{q_{O_2}^{\bullet}}_{Sr-top} + q_{O_2} e^{-} \right\}$ (O_2-adsorbed at Sr-top site)	$\mu_{O_2^{Sr-top}} = \mu^0_{O_2^{Sr-top}} + k_B T \ln\left(\Gamma_{O_2^{Sr-top}} \Big/ \left(1 - \Gamma_{O_2^{Sr-top}}\right)\right) + q_{O_2} E_F$
$O_2\left(Sr_{br} - O_{top}\right) = \left\{ \left(O_2\right)^{q_{O_2}^{\bullet}}_{Sr-br-Otop} + q_{O_2} e^{-} \right\}$ (O-O adsorbed together at Sr-Sr bridge and O-top sites)	$\mu_{O_2^{Sr-br-Otop}} = \mu^0_{O_2^{Sr-br-Otop}} + k_B T \ln\left(\Gamma_{O_2^{Sr-br-Otop}} \Big/ \left(1 - \Gamma_{O_2^{Sr-br-Otop}}\right)\right) + q_{O_2} E_F$
$O\left(O_{top}\right) = \left\{ O^{q_O^{\bullet}}_{O-top} + q_O e^{-} \right\}$ (O-adsorbed at O-top site)	$\mu_{O_{O-top}} = \mu^0_{O_{O-top}} + k_B T \ln\left(\Gamma_{O_{O-top}} \Big/ \left(1 - \Gamma_{O_{O-top}}\right)\right) + q_O E_F$

Supplementary Table 2: Possible reaction mechanisms of Oxygen Reduction on a model (001)-SrO terminated surface classified according to the type of site the O_2 is adsorbed, and type of site the adsorbed molecule dissociates. Symbols `-‘, ‘(*C*,*M*)’ and (*C*,*I*) are defined in “Methods” section in the main text. Several kinetically relevant mechanisms and also described in detail in Supplementary Table 3.

	Sr_{br} (*C*,*M*)	Sr_{top} (*C*,*M*)	O_{top}	V_O (*C*,*M*)	[$Sr_{br}O_{top}$] (*C*,*I*)	[$Sr_{br}Sr_{br}$] (*C*,*I*)	[Sr_bSr_{top}] (*C*,*I*)	[$Sr_{br}V_O$] (*C*,*I*)	[$Sr_{top}Sr_{top}$] (*C*,*I*)	[$Sr_{to}O_{top}$] (*C*,*I*)	[$Sr_{top}V_O$] (*C*,*I*)	[$O_{top}O_{top}$] (*C*,*I*)	[$O_{top}V_O$] (*C*,*I*)	[V_O-V_O] (*C*,*I*)
Sr_{br}	A1	A12	-	A23	A27	-	-	-	-	-	-	-	-	-
Sr_{top}	A2	A13	-	A24	A28	-	-	-	-	-	-	-	-	-
O_{top}	A3	A14	-	A25	A29	-	-	-	-	-	-	-	-	-
V_O	A4, A5	A15,A16	-	A26	A30, A31	-	-	-	-	-	-	-	-	-
[Sr_{br}-O_{top}]	-	A17	-	-	A32	-	-	-	-	-	-	-	-	-
[Sr_{br}-Sr_{br}]	-	A18	-	-	-	A33	-	-	-	-	-	-	-	-
[Sr_{br}-Sr_{top}]	-	-	-	-	-	-	A34	-	-	-	-	-	-	-
[Sr_{br}-V_O]	-	A19	-	-	-	-	-	A35	-	-	-	-	-	-
[Sr_{top}-	A6	-	-	-	-	-	-	-	A36	-		-	-	-
[Sr_{top}-O_{top}]	A7	-	-	-	-	-	-	-	-	A37		-	-	-
[Sr_{top}-V_O]	A8	-	-	-	-	-	-	-	-	-	A38	-	-	-
[O_{top}-O_{top}]	A9	A20	-	-	-	-	-	-	-	-	-	A39	-	-
[O_{top}-V_O]	A10	A21	-	-	-	-	-	-	-	-	-	-	A40	-
[V_O-V_O]	A11	A22	-	-	-	-	-	-	-	-	-	-	-	A41

Supplementary Table 3: Key mechanisms for oxygen reduction at the SrO-terminated surface of LSC cathode considered in this study. Various symbols are neutral building units as described in "Methods" section in the main text.

	Mech. A5	Mech. A9	Mech. A16	Mech. A20	Mech. A26	Mech. A39
	Adsorption(Sr_{br}) diffusion to s_{AO}	Adsorption(Sr_{br})dissociation on $2O_{top}$	Adsorption at (Sr_{top}), diffusion towards s_{AO}	Adsorption at (Sr_{top}), dissociation at $2O_{top}$	Adsorption at (V_O), dissociation at V_O	Dissociative adsorption at $2xO_{top}$
Step 1	null+O_2 = *O_2(Sr_{br})	null+O_2= *O_2(Sr_{br})	null+O_2 = *O_2(Sr_{top})	null+O_2= *O_2(Sr_{top})	null+O_2 +V_O= sO_2	null+O_2 = 2*O(O_{top})
Step 2	*O_2(Sr_{br})+ s_{AO} (far)= *O_2(Sr_{br})+ s_{AO} (near)	*O_2(Sr_{br})= 2*O(O_{top})	*O_2(Sr_{top})+s_{AO} (far)=*O_2(Sr_{top})+s_{AO}(near)	*O_2(Sr_{top})= 2*O(O_{top})	sO_2+ s_{AO} (far)= O(O_{top}) + s_{AO}	2*O(O_{top}) + 2s_{AO} = 2null
Step 3	*O_2(Sr_{br})+ s_{AO} (near) = *O(O_{top})	2*O(O_{top}) + 2s_{AO} = 2null	*O_2(Sr_{top})+ s_{AO} (near) = *O(O_{top})	2*O(O_{top}) + 2s_{AO} = 2null	*O(O_{top}) + s_{AO} = null	v = s_{AO} (x2)
Step 4	*O(O_{top}) + s_{AO} = null	v = s_{AO} (x2)	*O(O_{top}) + s_{AO} = null	v = s_{AO} (x2)	v = s_{AO} (x2)	
Step 5	v = s_{AO} (x2)		v = s_{AO} (x2)			
Net	O_2+ 2v = null	O_2+ 2v = null	O_2+ 2v = null	O_2+ 2v = null	O_2+ 2v = null	O_2+ 2v = null

1.2 CoO_2 Termination

Neutral building units of surface species of the CoO_2 termination and their chemical potentials are defined in Supplementary Table 4, and their molecular configurations in Figure 3 in the main text. Primary symbols O_2, V, and O refer to diatomic oxygen, bulk oxygen vacancies, and oxygen atoms, respectively. The subscripts 's', 'b' of O_s and O_b refer to location at a surface or bulk (in this case, oxygen site), respectively. The superscripts indicate species charge relative to a normal lattice oxygen ion and q_{ads}, q_{diss}, and q_{incorp} are the charge transferred in adsorption, dissociation, incorporation steps, respectively. In Supplementary Table 4, the unit 'v' denotes a bulk lattice site with

oxygen removed and 2 electrons added to the bulk. 's' denotes surface oxygen defect with $(q_S - q_O)$ electrons donated to the bulk. The unit 'sO_2' denotes an oxygen molecule chemisorbed (inserted) into a single surface vacancy in the CoO_2-termination, with $(q_{O_2} - q_O)$ electrons transferred to the bulk. The unit 'ss' denotes a surface site with two adjacent surface oxygens removed and $2(q_S - q_O)$electrons donated to the bulk. The unit 'CoO_2ss' denotes oxygen molecule adsorbed on top of surface Co near a divacancy (ss), and with electronic charge $(q_{O_2} + 2q_S - q_O)$ transferred from the bulk to the adsorbed molecule. The unit sO_2 +s(far) denotes O_2 chemisorbed and incorporated (barrier less) single isolated surface vacancy (a second vacancy is far from it). Similarly, CoO and CoO_2 respectively denote O-adatom and O_2-chemisorbed on a perfect surface lattice with no surface vacancies nearby. Supplementary Table 5 shows a matrix of various possible mechanisms of oxygen exchange on CoO_2 termination, based on the 8 possible sites of adsorption (rows) and 8 possible sites of dissociation (columns) on a CoO_2 terminated LSC surface. In all 12 distinct reaction pathways emerge on the CoO_2 surface, given in Supplementary Table 6 and Supplementary Table 7.

Supplementary Table 4: Definitions of neutral building units of the intermediates on (001)CoO_2-termination used in this study, and their chemical potentials. Itinerant electronic structure of the LSC material is assumed[1-4], wherein electrons are taken from and donated to the Fermi level.

Neutral Building Unit	Chemical potential
$v = \left\{V_{O_S}^{\bullet\bullet} + 2e^- - O_{O_S}^X\right\}$ (Isolated bulk vacancy)	$\mu_V = \mu_V^0 + k_B T \ln\left(x_V / \left(1 - x_V\right)\right) + 2E_F$
$s = \left\{V_{O_s}^{q_s^\bullet} + (q_s - q_o)e^- - O_{O_s}^{s\dot{O}}\right\}$ (Isolated surface vacancy)	$\mu_s = \mu_s^0 + k_B T \ln\left(\Gamma_s / (1 - \Gamma_s)\right) + (q_s - q_O)E_F$

$sO_2 = \left\{ (O_2)_{O_S}^{q_{sO_2}^{\bullet}} + (q_{O_2} - q_O)e^- - O_{O_S}^{q_O^{\bullet}} \right\}$ (ads-O_2 at surface vacancy)	$\mu_{sO_2} = \mu_{sO_2}^0 + k_B T \ln\left(\Gamma_{sO_2} / (1 - \Gamma_{sO_2})\right) + (q_{O_2} - q_O)E_F$
$ss = \left\{ (2V)_{O_S}^{2q_S^{\bullet}} + 2(q_S - q_O)e^- - 2O_{O_S}^{q_O^{\bullet}} \right\}$ (Surface di-vacancy)	$\mu_{SS} = \mu_{SS}^0 + k_B T \ln\left(\Gamma_{SS} / (1 - \Gamma_{SS})\right) + 2(q_S - q_O)E_F$
$CoO_2ss = \left\{ \begin{array}{l} (O_2)_{Co}^{q_{CoO_2ss}^{\bullet}} + (2V)_{O_S}^{2q_S^{\bullet}} \\ + (q_{O_2} + 2q_S - 2q_O)e^- - 2O_{O_S}^{q_O^{\bullet}} \end{array} \right\}$ (O_2-chemisorbed near surface di-vacancy)	$\mu_{CoO_2ss} = \mu_{CoO_2ss}^0 + k_B T \ln\left(\Gamma_{CoO_2ss} / (1 - \Gamma_{CoO_2ss})\right)$ $+ (q_{O_2} + 2q_S - 2q_O)E_F$
$CoO_2s + s(far) = \left\{ \begin{array}{l} (O_2)_{Co}^{q_{CoO_2s}^{\bullet}} + 2V_{O_S}^{q_S^{\bullet}} \\ + (q_{O_2} + 2q_S - 2q_O)e^- - 2O_{O_S}^{q_O^{\bullet}} \end{array} \right\}$ (O_2-chemisorbed near an isolated surface vacancy)	$\mu_{CoO_2s+s} = \mu_{CoO_2s+s}^0 + k_B T \ln\left(\Gamma_{CoO_2s+s} / (1 - \Gamma_{CoO_2s+s})\right)$ $+ (q_{O_2} + 2q_S - 2q_O)E_F$
$CoO = \left\{ O_{Co}^{q_O^{\bullet}} + q_O e^- \right\}$ (O-chemisorbed on surface Co)	$\mu_{CoO} = \mu_{CoO}^0 + k_B T \ln\left(\Gamma_{CoO} / (1 - \Gamma_{CoO})\right) + q_O E_F$
$CoO_2 = \left\{ (O_2)_{Co}^{q_{CoO_2}^{\bullet}} + q_{O_2} e^- \right\}$ (O_2-chemisorbed on surface Co)	$\mu_{CoO_2} = \mu_{CoO_2}^0 + k_B T \ln\left(\Gamma_{CoO_2} / (1 - \Gamma_{CoO_2})\right) + q_{O_2} E_F$

Supplementary Table 5: Possible reaction mechanisms of Oxygen Reduction on a model CoO_2 terminated surface classified according to the type of site the O_2 is adsorbed, and type of site the adsorbed molecule dissociates. Symbols `-‘, (*C,M*) and (C,I) are defined in “Methods” section in the main text. All 12 mechanisms that are studied are indicated and also described in detail in Supplementary Table 6, Supplementary Table 7.

Adsorbs at / Dissociates on	Co (*C*,*M*)	$V_{\ddot{O}}$ (*C*,*I*)	O	[$V_{\ddot{O}}$-$V_{\ddot{O}}$] (*C*,*I*)	[Co-Co] (*C*,*I*)	[Co-O] (*C*,*I*)	[Co-$V_{\ddot{O}}$] (*C*,*I*)	[O-$V_{\ddot{O}}$] (*C*,*I*)	[O-O] (*C*,*I*)
Co	B6	B3	-	-	-	-	-		-
$V_{\ddot{O}}$	B4	B1	-	-	-	-	-		-
O	B5	-	-	-	-	-	-		-
[$V_{\ddot{O}}$-$V_{\ddot{O}}$]	B2	-	-	B7	-	-	-		-
[Co-Co]	-	-	-	-	B8	-	-		-
[Co-O]	-	-	-	-	-	B9			-
[Co-$V_{\ddot{O}}$]	-	-	-	-	-	-	B10		-
[O-$V_{\ddot{O}}$]	-	-	-	-	-	-		B11	
[O-O]	-	-	-	-	-	-	-	-	B12

Supplementary Table 6: Mechanisms B1-B6 for oxygen reduction at the CoO_2-terminated surface of LSC cathode considered in this study. Various symbols are neutral building units as described in "Methods" section in the main text.

	Mech.B1	Mech. B2	Mech. B3	Mech. B4	Mech. B5	Mech. B6
	Chemisorption at vacancy (s), dissociation at 's'	Chemisorption at Co, dissociation at nearby 'ss'	Chemisorption at vacancy (s), dissociation at 's'	Chemisorption at Co, dissociation at nearby 's'	Chemisorption at Co, dissociation at 'O_S'	Chemisorption at Co, dissociation at 'Co'
Step 1	s + O_2 = sO_2	ss + O_2 = CoO_2ss	O_2 + s + s = sO_2 + s (far)	O_2 + s + s = CoO_2 + 2s (far)	O_2 + null = CoO_2	O_2 + null= CoO_2
Step 2	sO_2 + s(far) = sO_2 +s(near)	CoO_2ss= null	sO_2 + s(far) = CoO +s(near)	CoO_2 +2s(far) = CoO_2 + s(near) + s(far)	CoO_2 = CoO + sO_2	CoO_2 = 2CoO
Step 3	sO_2 +s(near) = null	s+s = ss	CoO +s(near) = null	CoO_2 + s(near) + s(far) = CoO +s	CoO + sO_2+ 2s = 2CoO+2s(near) (hopping)	2CoO +2s(far) = 2CoO+2s(near) (hopping)
Step 4	v = s (x 2)	v = s (x 2)	v = s (x 2)	CoO +s =null (x 2) (hopping)	2CoO +2s(near) = 2null	2CoO +2s(near) = 2null
Step 5				v = s (x 2)	v = s (x 2)	v = s (x 2)
Net	O_2+ 2v = null	O_2+ 2v = null	O_2+ 2v = null	O_2+ 2v = null	O_2+ 2v = null	O_2+ 2v = null

Supplementary Table 7: Mechanisms B7-B12 for oxygen reduction at the BO_2-terminated surface of LSC cathode considered in this study. Symbols v, s, ss, sO_2, CoO_2ss, null are neutral building units as described in "Methods" section in the main text.

	Mech. B7	Mech. B8	Mech. B9	Mech. B10	Mech. B11	Mech. B12
	Dissociative chemisorption at 'ss'	Dissociative chemisorption at [Co-Co]	Dissociative chemisorption at [Co-O]	Dissociative chemisorption at [Co-Vö]	Dissociative chemisorption at [O-Vö]	Dissociative chemisorption at [O-O]
Step 1	O_2 + ss= null	O_2 +null = 2CoO	O_2 +null=CoO + sO_2	O_2 + s =CoO	O_2 + s = sO_2	O_2 + null = $2sO_2$
Step 2	s+s = ss	2CoO +2s = 2null	CoO+sO_2 +2s = 2null	CoO + s = null (hopping)	sO_2 + s= CoO +s	$2sO_2$ = 2CoO
Step 3	v = s (x 2)	v = s (x 2)	v = s (x 2)	v = s (x 2)	CoO +s =null (hopping)	2CoO + 2s = 2null
Step 4					v = s (x 2)	v = s (x 2)
Net	O_2+ 2v = null	O_2+ 2v= null	O_2+ 2v= null	O_2+ 2v= null	O_2+ 2v = null	O_2+ 2v= null

Supplementary Table 8 Thermodynamic and kinetic model parameters. List of thermodynamic (binding, formation, vibration energies, O_2 reference, etc.) and kinetic (k_{ads}, β, energy barriers) model parameters used in this work for all relevant mechanisms of SrO and CoO_2 terminations of LSC-50.

Name	Description	Value	Comments / Reference
O_2 gas parameters at 650°C			
Θ_{vib}	O-O vibrational mode	2244 K	Reference [5]
$G_{O_2}^{vib}$(T)	Vibrational free energy from O-O stretch mode at 650°C	8.94×10^{-2} eV	
$P_{O_2}^{gas}/(N_0\sqrt{2\pi m k_B T})$	O_2 physisorption rate at T=650 °C, pO_2 =0.2 atm	4.96×10^{7} #O_2/Co/s (4.97×10^{2} moles/m^2s)	Reference [6]
$q_{2D\text{-}gas}$	O_2 translational partition function	1558.80 (unitless)	
Θ_{rot} , q_{rot}	O_2 rotational mode, partition function	2.08K, 299.99 (unitless)	Reference [7]
$G_{O_2}^{rotation}$(T)	Rotational free energy of O_2 molecule at 650 °C	-0.43 eV	Calculated
$G_{O_2}^{rot-vib}$(T)	Roto-vibrational free energy of O_2 molecule at 650 °C	-0.34 eV	Calculated
k_{ads}^{M}, k_{ads}^{I}	Pre-exponential factors for O_2 adsorption at 650 °C	k_{ads}^{I} = 0.65 m-s/kg, k_{ads}^{M} =17.55 m-s/kg	"Methods" section

Bulk LSC-50 parameters			
a	Bulk vacancy interaction parameter	54.0 kcal mol^{-2} (2.34eV)	Constant for LSC-50[2]
Δh_O^0	Standard enthalpy change between gas and bulk-O	-16.9 kcal mol^{-1}	Constant for LSC-50[2]
ΔS_O^0	Standard entropy change between gas and bulk-O	-15.4 kcal mol^{-1}	Constant for LSC-50[2]
$x_{v,bulk}$(oxygen vacancy in bulk LSC-50)	Ideal, non-configurational vacancy formation enthalpy (Δh^0) for bulk vacancy at 650 °C (DFT only, with correction for finite vacancy concentration, from ref. [2])	1.02 eV, 0.84 eV, 0.82 eV [2]	Calculated
q_{latt}^{vib}	Lattice-O vibrational partition function (3 modes of $\theta_{Einstein}$=275 K)	3.34 (unitless)	"Methods" Section for the explanation of 275K
E_F	Fermi-level change (eV) due to addition/subtraction of electrons[8]	$E_F = 1.5 \times a \times x_v$	Reference
Sr-rich AO-termination (SrO termination) parameters			
Γ_s(vacancy on SrO)	Vacancy formation energy (DFT only, at 0K)	1.48 eV	value from DFT
	Site fraction at 650 °C, 0.2 atm pO_2	6.1×10^{-5} (per Co)	Calculated
$*O_2(Sr_{br})$	Reaction energy (DFT only, with vibrational energy at 650 °C and corrections[§])	-0.87 eV, -0.40 eV	$O_2 + *(Sr_{br}) \rightarrow *O_2(Sr_{br})$
	Charge (q_{O2})	$1e^-$	$*O_2^{1-}$
	Site fraction at 650 °C, 0.2 atm pO_2	4.7×10^{-7}(per Co)	Calculated
$*O_2(Sr_{top})$	Reaction energy (DFT only, with vibrational energy at 650 °C and corrections[§])	0.82 eV,-0.35 eV	$O_2 + *(Sr_{top}) \rightarrow *O_2(Sr_{top})$
	Charge (q_{O2})	$1e^-$	$*O_2^{1-}$
	Site fraction at 650 °C, 0.2 atm pO_2	1.56×10^{-7} (per Co),	Calculated
$*O_2(Sr_{br}\text{-}O_{top})$	Reaction energy (DFT only, with vibrational energy at 650 °C and corrections[§])	-1.11 eV, -0.23 eV	$O_2 + *(Sr_{br}\text{-}O_{top}) \rightarrow *O_2(Sr_{br}\text{-}O_{top})$
	Charge (q_{O2})	$1e^-$	$*O_2^{1-}$
	Site fraction at 650 °C, 0.2 atm pO_2	5.0×10^{-7} (per Co), see Fig. 2	Calculated
$*O(O_{top})$	Reaction energy (DFT only, with vibrational energy at 650 °C and corrections[§])	-2.12 eV, -1.45 eV	$\frac{1}{2} O_2 + *(O_{top}) \rightarrow *O(O_{top})$
	Charge (q_O)	$1e^-$	$*O_2^{1-}$
	Site fraction at 650 °C, 0.2 atm pO_2	2.3×10^{-5} (per Co),	Calculated
β	kinetic symmetry parameter	See Table 2 in main text	
kinetic barriers		See Fig. 3 in main text	
CoO_2 termination parameters			
Γ_s(vacancy on CoO_2)	Vacancy formation energy (DFT only, at 0 K)	$5.48 \text{ eV} \times \Gamma_s - 0.43$ eV	Calculated

			Γ_s is surface oxygen vacancy concentration
	Site fraction at 650 °C, 0.2 atm pO_2	0.28 (per surface-O)	Calculated
$*CoO_2$	Reaction energy (DFT only, with vibrational energy at 650 °C and corrections[§])	-1.10 eV, -0.40 eV	O_2 +s +s +*(Co) → CoO_2s+s(far)
	Charge (q_{O2})	0.5 e^-	$*O_2^{0.5-}$
	Site fraction at 650 °C, 0.2 atm pO_2	2.21×10^{-12}(per Co),	Calculated
$*CoO_2$ss	Reaction energy (DFT only, with vibrational energy at 650°C and corrections[§])	-1.72 eV, -1.02 eV	O_2 +ss(divacancy) + *(Co) → CoO_2ss
	Charge (q_{O2})	2 e^-	$*O_2^{0.5-}$
	Site fraction at 650 °C, 0.2 atm pO_2	7.02×10^{-7}(per Co),	Calculated
$*O_{O\text{-}top}$+s(far)	Reaction energy (DFT only, with vibrational energy at 650 °C and corrections[§])	-1.82 eV, -1.12 eV	O_2 +s +s +*(Co) → $O_{O\text{-}top}$+s(far)
	Charge (q_{O2})	1e^-	$*O_2^{1-}$
	Site fraction at 650 °C, 0.2 atm pO_2	2.4×10^{-5} (per Co),	Calculated
$*sO_2$+s(far)	Reaction energy (DFT only, with vibrational energy at 650 °C and corrections[§])	-2.04 eV, -1.34 eV	O_2 +2s → sO_2+s(far)
	Charge (q_{O2})	2 e^-	$*O_2^{2-}$
	Site fraction at 650 °C, 0.2 atm pO_2	4.3×10^{-4} (per Co)	Calculated
CoO+s(far)	Reaction energy (DFT only, with vibrational energy at 650 °C and corrections[§])	-1.97 eV, -1.97 eV	O_2 +2s + *(Co)→ CoO+s(far) (one vacancy is filled)
	Charge (q_O)	2e^-	$*O^{2-}$
	Site fraction at 650 °C, 0.2 atm pO_2	0.25 (per Co)	Calculated
CoO+s(near)	Reaction energy (DFT only, with vibrational energy at 650 °C and corrections[§])	-1.99 eV, -1.99 eV	O_2 +2s + *(Co)→ CoO+s(near) (one vacancy is filled)
	Charge (q_O)	2 e^-	$*O^{2-}$
	Site fraction at 650 °C, 0.2 atm pO_2	0.25 (per Co)	Calculated
2CoO+2s(far)	Reaction energy (DFT only, with vibrational energy at 650 °C and corrections[§])	0.01 eV, 0.01 eV	O_2 +2s +2*(Co)→ CoO+CoO+s+s(far) (vacancies not filled)
	Charge (q_O)	2e^-	$*O_2^{2-}$
	Site fraction at 650 °C, 0.2 atm pO_2	8.90×10^{-11} (per Co)	Calculated
CoO+sO_2+2s(far)	Reaction energy (DFT only, with vibrational energy at 650 °C and corrections[§])	0.23 eV, 1.46 eV	O_2 +2s +*(Co)→ [CoO+sO_2]+s+s(far)
	Charge (q_O)	2e^-	$*O_2^{2-}$
	Site fraction at 650 °C, 0.2 atm pO_2	6×10^{-18} (per Co)	Calculated
β	kinetic symmetry parameters	See Table 2 in main text	

$\Delta G_{f,i}^{0}$	kinetic barriers	See Fig. 3 in main text	

§Vibrational energies of adsorbed species ($^*O_2$ or $^*O$) and O_2 gas molecule are included, translational modes of O_2 gas are not included. Configurational entropy is not included. Site fractions are calculated by using the scheme given in "Methods" Section and by constructing equations similar to equation (11) in main text.

Supplementary Table 9: List of assumptions regarding modes of molecular vibrations for calculating vibrational free energy of surface species, in order to calculate their equilibrium concentrations, following the method described in the Methods.

Surface	Type of adsorbate	Neutral building units	Modes of vibration
CoO_2 termination of LSC-50	*O adsorbed on Co	CoO+s(far), CoO+s(near)	3 modes with θ_E = 275 K
	$^*O_2$ adsorbed on Co	CoO_2+2s, CoO_2s+s(far), CoO_2ss	5 modes with θ_E = 275 K, 1 O-O stretching mode (2244 K) from gas
	$^*O_2$ inserted into vacancy	sO_2+s(far), sO_2+s(near)	5 modes with θ_E = 275 K, 1 O-O stretching mode (2244 *K*) from gas
SrO-termination of LSC-50	*O adsorbed on surface site	$^*O(O_{top})$, $^*O(Sr_{top})$, $^*O(Sr_{br})$	2 modes with θ_E = 275 K, 1 O-O stretching mode (2244 *K*) from gas
	$^*O_2$ losely bound on surface	$^*O_2(Sr_{br})$, $^*O_2(Sr_{top})$	3 modes with θ_E = 275 K, 3 modes of O_2 molecule's roto-vibration
	$^*O_2$ strongly bound on surface	$^*O_2(Sr_{br}\text{-}O_{top})$	4 modes with θ_E = 275 K, 2 O-O stretching modes (2244 K) from gas
	$^*O_2$ strongly bound into vacancy	$^*O_2(O_{top})$ (O_2 inserted into s_{AO})	5 modes with θ_E = 275 K, 1 O-O stretching mode (2244 K) from gas

2 Suppelementary Note 2

2.1 Calculating rates from DFT and other Energetics

Here we will describe the equations used to calculate energetics of various ORR mechanisms using DFT, taking Mechanism B3 for example. Using equation (2) of main text, the exchange rate of the dissociation (O_2 insertion into vacancy) step of Mechanism B3 can be expressed as,

$$\begin{aligned} R_0 &= k_{diss}\Gamma_{CoO_2S}\exp\left(\frac{-\Delta G^0_{f,diss}+(1-\beta)q_{diss}E_F}{k_BT}\right) \\ &= \frac{k_{diss}P^{gas}_{O_2}}{f^{solid}_{O_2}}\exp\left(\frac{1}{k_BT}\begin{pmatrix}\left(\Delta G^0_{ads}-q_{diss}E_F\right) \\ -\Delta G^0_{f,diss}+(1-\beta)q_{diss}E_F\end{pmatrix}\right) \end{aligned} \quad (1)$$

where k_{diss} is the pre-exponential factor of dissociation step (taken as k_BT/h), Γ_{CoO_2S} is the concentration of adsorbed O_2 with nearest-neighbor surface vacancies, ΔG^0_{ads} is the non-configurational Gibb's free energy change during the adsorption step, q_{diss} is the electron transferred to the oxygen during dissociation (using DFT we estimate, $q_{\text{diss}}=1$), β is the reaction symmetry parameter, $\Delta G^0_{f,diss}$ is the reaction barrier estimated using DFT. The Fermi-energy shift E_F measures the change in electronic energy due to the filling of oxygen vacancies. ΔG^0_{ads} does not include translational free energy from O_2 gas or configurational entropy of the adsorbate; and is an example of local, non-configurational Gibb's free energy change. Local non-configurational free energy is denoted as ΔG^*. To obtain ΔG^* at SOFC conditions, we use the same methodology as implemented by Lee et al.[9,10], where thermodynamic terms, i.e., of solid phase vibrations, are added into the

zero-temperature DFT+U energetics. Our approximation in the treatment of vibrational free energy is that we assume the vibrational free energy of the solid phase atoms that are not involved in the reaction do not change significantly during the reaction[9,10]. For an adsorption reaction, A_{gas} + * → *A, the ΔG^* for adsorption step ($=\Delta G^0_{ads}$), is described as

$$\Delta G^0_{ads} = \Delta G^* = \left(E^{VASP}_{*O_2} - E^{VASP}_{*} - E^{VASP}_{O_2,gas} + E^{don}_{DFT}\right) + \left(G^{vib}_{*O_2}(T) - G^{roto-vib}_{O_2,gas}(T)\right) \qquad (2)$$

where $E^{VASP}_{*O_2}$, E^{VASP}_{*}, $E^{VASP}_{O_2,gas}$ are the 0K energy of DFT supercell with adsorbed species (example: CoO_2s) with the vacant site (denoted as *, such as a surface Co near a vacancy s on CoO_2 termination), and isolated O_2 molecule, respectively. Term E^{don}_{DFT} is the electron donation correction energy, needs to be added to remove the effect of simulating the DFT energy at finite supercell size, as explained in "Methods" section in the main text. $G^{vib}_{*O_2}(T)$, $G^{roto-vib}_{O_2,gas}(T)$are, respectively, the vibrational free energy of the oxygen in adsorbate (CoO_2s), and the roto-vibrational contribution from O_2 molecule (values in Table 1 in main text). Vibrational energy of 2 oxygens of CoO_2s is calculated with degrees of freedom from solid phase (Einstein temperature of 275K, see "Methods" section in the main text), one degree of freedom taken as O-O gas stretching mode. Finally, reaction

barrier $\Delta G^0_{f,i}$ is calculated using cNEB methods embedded in VASP[11,12]. Appropriate DFT corrections were applied in "Methods" section in the main text.

2.1.1 Example of rate calculation

At SOFC conditions of 650°C, 0.2 atm pO_2, rates (R_0, #O_2/Co-site/second) of individual steps of Mech.B3 in order-of-magnitude approximation are as follows,

<u>Step 1</u> This step is O_2 adsorption on Co near a surface vacancy and then insertion into a nearby vacancy. This step is barrierless and requires a fixed O-O orientation for insertion. The equation for R_0 is

$$R_0(step1) = (k^I_{\mathrm{ads}} P^{gas}_{O_2}) \times (2 \times 2\Gamma_s \times (1 - \Gamma_{Co+s}))$$

R_0= (0.65×0.2×101325)×(2×2×0.28×0.75) = 1.1×10^4 (#O_2/Co-sec), becomes rate-limiting for this mechanism at 650°C and 0.2atm pO_2. Factor 2 (in $2\Gamma_s$) accounts for the fact that for an oxygen vacancy site-fraction of Γ_s, $2\Gamma_s$ vacancies are available per Co, furthermore, 2 equivalent Co sites are available for O_2 adsorption per such surface vacancy. A significant amount of surface Co is predicted to be covered with adsorbed O (Γ_{CoO+S}=0.25 (site fraction per Co) at 650°C, 0.2 atm pO_2), hence available sites need to be adjusted by the factor $(1-\Gamma_{CoO+S})$. The insertion step requires a fixing O-O orientation, so we are using the immobile pre-exponential factor. Details of calculation of pre-exponential factor (k^I_{ads}) are given in "Methods" section in the main text. For this reaction step, there is another possibility for reaching the final state of CoO_2s+s(far), where O_2 adsorbs on a Co that is distant from a vacancy, and then diffuses towards a

vacancy. Since the concentration of such species (CoO_2+2s(far)) is predicted to be small (see Figure 3 in the main text and Table 1 in the main text for details), this path is not likely to be fast enough.

Step 2 This step involves diffusion of *O (from sO_2) towards a second surface vacancy (s) via Co-O-Co path (barrier 0.6eV). The expression for R_0 is:

$$R_0\left(diffusion\right)=\left(8\times D_V\times 2\Gamma_S\times\Gamma_{sO_2}\right)/d^2$$

where D_V is the diffusivity of surface *O, Γ are the concentrations of vacancy and *O, and d is a hop distance. Details of this derivation can be found in Supplementary Information from the work of Mastrikov et al.[13]. The rate is calculated as $R_0=8\times(8\times10^{-10}m^2/s)\times2\times0.28\times(5.2\times10^{-4})\times(2.68\times10^{19}m^2) = 4.12\times10^7$ (#O_2/Co-sec), which is faster than steps 1 and 2. *O will diffuse towards the vacancy up to its nearest neighbor position (O adatom on Co) and then incorporate into the vacancy (step 4) as follows. There is a parallel path where oxygen vacancy diffuses from bulk to surface near a *O. The bulk oxygen diffusion barrier for LSC-50 is close to 1eV (Table II of Haar et al.[14]). This rate is $R_0=\left(k_BT/h\right)\times x_V\times\Gamma_{O_{ads}}\times\exp\left[-1/k_BT\right]$=$(1.92\times10^{13})\times(3.12\times10^{-2})\times(5.2\times10^{-4})\times(3.45\times10^{-6})$=1075 (#$O_2$/Co-sec).This path is much slower than surface diffusion, so we can combine this path with the surface diffusion path. For the AO surface rate limiting step,

we also get surface diffusion path much faster than the path where the bulk oxygen vacancy diffuses to the surface *O.

Step 3**:** This step involves the incorporation of species *O (species CoO+s(near)) into the nearby surface vacancy, with a 0.4eV barrier. The expression for R_0 is:

$$R_0(step4) = (k_B T/h)\Gamma_{CoO+s(near)} \exp\left[\left(-0.4 + (1-\beta)q_{incorp}E_F\right)/k_B T\right].$$

O^{2-} of CoO has charge of 2- hence there is no charge transfer ($q_{\text{incorp}} = 0$) in this step, and R_0=(1.92×10^{13})×0.25×6.54×10^{-3} = 3.14×10^{10} (#O_2/Co-sec) which is faster than steps 1,2.

Finally Step 4, involves diffusion of surface oxygen towards the bulk LSC-50, through the diffusion of bulk vacancies. Bulk oxygen diffusion barrier for LSC-50 is close to 1eV (Table II of Haar et al.[14]). Hence the value for R_0 of bulk diffusion of oxygen via bulk oxygen vacancies will approximately be $(k_B T/h) \times x_V \times \exp[-1/k_B T]$ = (1.92×10^{13})×(3.12×10^{-2})×(3.45×10^{-6}) = 2.06×10^{6} (#O_2/Co-sec) at 650°C, 0.2 atm pO_2. Thus the R_0 for bulk diffusion is faster than the rate-limiting step of oxygen adsorption (step 1 above).

2.2 Error estimation for DFT energetics

In this section we discuss the various sources of error and the extent to which they may affect the predicted results. Let us take the example of mechanism A9 on SrO surface, with the surface diffusion, being the rate limiting step. The overall rate of this mechanism is proportional to, $R_0 = 8D_v\Gamma_{Oads}\Gamma_{Ov}/d^2 = 8D_0 e^{-\Delta E_{\text{dif}}/k_B T}\Gamma_{Oads}\Gamma_{Ov}/d^2$ where D_v is the surface

oxygen diffusivity. ΔE_{dif} is the surface oxygen vacancy diffusion barrier. Γ_{Oads} and Γ_{Ov} is the concentration of reactant (*O and surface VO). Γ_{Oads} and Γ_{Ov} are 10^{-5} to 10^{-6}, which is very small. So we can write them in simple equation,

$$\Gamma_{Oads} \approx \frac{\Gamma_{Oads}}{1-\Gamma_{Oads}} = \frac{x_v}{1-x_v} \bullet e^{\frac{-\Delta G^0_{ads}}{k_B T}} \tag{3}$$

$$\Gamma_{Ov} \approx \frac{\Gamma_{Ov}}{1-\Gamma_{Ov}} = \frac{x_v}{1-x_v} \bullet e^{\frac{-\Delta G^0_{sOv}}{k_B T}} \tag{4}$$

where ΔG^0_{ads} is the formation energy of reactant (*O) with all corrections. $-\Delta G^0_{sOv}$ is the surface oxygen vacancy segregation energy. x_v is the bulk oxygen vacancy concentration from Mizusaki's experiment[15]. So we can write the log(R_0) as,

$$\log R_0 = \frac{-\Delta E_{\text{dif}}}{k_B T} + \frac{-\Delta G^0_{ads}}{k_B T} + \frac{-\Delta G^0_{sOv}}{k_B T} + c \tag{5}$$

where c is a constant. Thus, we can consider the standard deviation of log(R_0) as consisting of contributions from standard deviations of three DFT energy calculation. By the quadrature error rule, the standard deviation of log(R_0) can then be written as,

$$S_N(\log R_0) = \sqrt{\left(\frac{S_{\Delta E_{\text{dif}}}}{k_B T}\right)^2 + \left(\frac{S_{\Delta G^0_{ads}}}{k_B T}\right)^2 + \left(\frac{S_{\Delta G^0_{sOv}}}{k_B T}\right)^2} \tag{6}$$

where $S_{\Delta E_{\text{dif}}}$, $S_{\Delta G^0_{ads}}$ and $S_{\Delta G^0_{sOv}}$ are the standard deviations of the three DFT energies. Typical ranges of errors in DFT defect formation free energies, binding free energies and reaction barriers are approximately 0.1 eV to 0.2 eV per reacting atom, which we average

to 0.15 eV to have a single value. We assume this range equals two times standard deviation of the relevant DFT energy, i.e., S_{DFT} = 0.075 eV. At 650℃, using supplementary equation (6) we get $S_N(\log R_0)$ =1.4. Thus the DFT-estimated reaction rates in this work, including that of the AO rate limiting reaction, could readily have an error bar as large as ±1.4 log units. This error bar is perhaps somewhat larger than, but similar to, the uncertainty in the experimentally-measured exchange rate. Jacob's et. al.[16] summarized the spreads in experimental *k** values and found 0.91 to 1.63 mean squared errors in log units. Furthermore, we see a spread of 1 to 1.5 log units in the measurements of k* for $La_{0.5}Sr_{0.5}CoO_3$ (see Figure 8 in the main text). While these are large errors, one of our key results, which is that the R_0 for the CoO_2 surface is 2-3 orders of magnitude larger than for the SrO surface (refer to Figure 8 in the main text), is at least somewhat larger than these uncertainties. Furthermore, we expect that errors in different reaction rates are likely to be correlated, and therefore relative values, like rates on two different surfaces, will likely have significantly smaller errors than any given rate. Thus, even with the error bars on the DFT predicted reaction rates, we still expect the SrO surface termination to offer slower ORR compared to the CoO_2 termination.

3 Supplementary References

1 Adler, S. B., Chen, X. Y. & Wilson, J. R. Mechanisms and rate laws for oxygen exchange on mixed-conducting oxide surfaces. *Journal of Catalysis* **245**, 91-109, doi:http://dx.doi.org/10.1016/j.jcat.2006.09.019 (2007).

2 Mizusaki, J., Mima, Y., Yamauchi, S., Fueki, K. & Tagawa, H. Nonstoichiometry of the perovskite-type oxides La1-xSrxCoO3-[delta]. *Journal of Solid State Chemistry* **80**, 102-111 (1989).

3 Lankhorst, M. H. R., Bouwmeester, H. J. M. & Verweij, H. Use of the Rigid Band Formalism to Interpret the Relationship between O Chemical Potential and Electron Concentration in La_{1-x}Sr_{x}CoO_{3- delta }. *Physical Review Letters* **77**, 2989 (1996).
4 Lankhorst, M. H. R., Bouwmeester, H. J. M. & Verweij, H. Thermodynamics and Transport of Ionic and Electronic Defects in Crystalline Oxides. *Journal of the American Ceramic Society* **80**, 2175-2198, doi:10.1111/j.1151-2916.1997.tb03107.x (1997).
5 Herzberg, G. *Moleculas Spectra and Molecular Structure 1: Spectra of Diatomic Molecules*. (van Nostrand Reinhold company, 1963).
6 Chorkendorff, I. & Niemantsverdrie, J. W. *Concepts of Modern Catalysis and Kinetics*. 79-128 (Wiley, 2003).
7 McQuarrie, D. A. *Statistical Mechanics*. 1 edn, (University Science Books, 2000).
8 Adler, S. B. Mechanism and kinetics of oxygen reduction on porous La1-xSrxCoO3-[delta] electrodes. *Solid State Ionics* **111**, 125-134 (1998).
9 Lee, Y.-L., Kleis, J., Rossmeisl, J. & Morgan, D. Ab initio energetics of LaBO_{3}(001) (B=Mn , Fe, Co, and Ni) for solid oxide fuel cell cathodes. *Physical Review B* **80**, 224101 (2009).
10 Lee, Y.-L. & Morgan, D. Ab initio and empirical defect modeling of LaMnO3+/-[small delta] for solid oxide fuel cell cathodes. *Physical Chemistry Chemical Physics* **14**, 290-302, doi:10.1039/c1cp22380a (2012).
11 Kresse, G. & Furthmüller, J. Efficient iterative schemes for ab initio total-energy calculations using a plane-wave basis set. *Phys. Rev. B* **54**, 11169 (1996).
12 Kresse, G. & Hafner, J. Ab initio molecular dynamics for liquid metals. *Phys. Rev. B* **47**, 558 (1993).
13 Mastrikov, Y. A., Merkle, R., Heifets, E., Kotomin, E. A. & Maier, J. Pathways for Oxygen Incorporation in Mixed Conducting Perovskites: A DFT-Based Mechanistic Analysis for (La, Sr)MnO3-d. *The Journal of Physical Chemistry C* **114**, 3017-3027, doi:10.1021/jp909401g (2010).
14 Stoerzinger, K. A. *et al.* Oxygen electrocatalysis on (001)-oriented manganese perovskite films: Mn valency and charge transfer at the nanoscale. *Energy & Environmental Science* **6**, 1582-1588, doi:10.1039/c3ee40321a (2013).
15 Mizusaki, T. K. J. S. M. S. A. K. K. Y. Y. N. J. Determination of oxygen vacancy concentration in a thin film of La0. 6Sr0. 4CoO3− δ by an electrochemical method. *Journal of The Electrochemical Society* (2002).
16 Jacobs, R., Mayeshiba, T., Booske, J. & Morgan, D. Material Discovery and Design Principles for Stable, High Activity Perovskite Cathodes for Solid Oxide Fuel Cells. *Advanced Energy Materials* (2018).
